# Water and soil contaminated by arsenic: the use of microorganisms and plants in bioremediation

**DOI:** 10.1007/s11356-021-17817-4

**Published:** 2021-12-02

**Authors:** Philippe N. Bertin, Simona Crognale, Frédéric Plewniak, Fabienne Battaglia-Brunet, Simona Rossetti, Michel Mench

**Affiliations:** 1 Génétique Moléculaire, Génomique et Microbiologie, UMR7156 CNRS - Université de Strasbourg, Strasbourg, France; 2 Water Research Institute, National Research Council of Italy (IRSA - CNR), Rome, Italy; 3 Environmental Biogeochemistry and Water Quality Unit, BRGM, Orléans, France; 4 Univ. Bordeaux, INRAE, BIOGECO, F-33615 Pessac, France

**Keywords:** Arsenic, Bioremediation, Phytoremediation, Microbial genomics, Metabolism, Phytomanagement

## Abstract

Owing to their roles in the arsenic (As) biogeochemical cycle, microorganisms and plants offer significant potential for developing innovative biotechnological applications able to remediate As pollutions. This possible use in bioremediation processes and phytomanagement is based on their ability to catalyse various biotransformation reactions leading to, e.g. the precipitation, dissolution, and sequestration of As, stabilisation in the root zone and shoot As removal. On the one hand, genomic studies of microorganisms and their communities are useful in understanding their metabolic activities and their interaction with As. On the other hand, our knowledge of molecular mechanisms and fate of As in plants has been improved by laboratory and field experiments. Such studies pave new avenues for developing environmentally friendly bioprocessing options targeting As, which worldwide represents a major risk to many ecosystems and human health.

## Introduction

Arsenic (As), which is often considered a non-essential element except for some organisms (but would be in fact an ultratrace element) and classified as a carcinogen, is a common contaminant of water, soil and food, and a global danger to human health (Nordstrom 2002; Zhao et al. 2010; ATSDR 2017; Marchant et al. 2017; da Silva et al. 2018a). Drinking water being considered as the main source of As ingestion, WHO recommended 10 µg L^−1^ as the As drinking water guideline value (WHO 2017), which has been adopted as the concentration limit in drinking water in most countries. Only a few guideline values for As concentrations in foodstuffs were defined. In Europe (EU 2015/1006), As concentration limits are given for some rice products (from 0.1 to 0.3 mg kg^−1^ wet weight). An acute or chronic exposure to As excess can cause many diseases, including various cancer forms. Its presence in water is one main source of contamination, but many studies have demonstrated its presence in fishes and crops from contaminated areas (Carlin et al. 2016; Jackson et al. 2012; Manjón and Ramírez-Andreotta 2020; Molin et al. 2015; WHO 2011; Zhao 2020). In several Chinese provinces, As-contaminated farmland impairs rice production (Li et al. 2019b). Irrigation of agricultural soils with As-rich water may contribute to As accumulation in soil and crops, and its entry into the food chain (Sandhi et al. 2018).

From natural or anthropogenic sources, As is present worldwide in the environment and many areas have a high As geochemical background suffering from soil and groundwater contamination, e.g. Chaco-Pampean plain in Argentina, West Bengal in India, Bangladesh, South-East Asia, and Limousin in France (Singh et al. 2015; Marchant et al. 2017; Antoni et al. 2019). Soil As contamination oftenly increases due to anthropogenic activities, e.g. As wood preservatives and treated wood washings, glassworks and crystal, mining and tailings, smelting, semiconductors, electronics and batteries, paints, toner, weapons, adhesives, disposal of industrial effluents, fossil fuel combustion, pesticides, and P fertilisers (Belon et al. 2012; Singh et al. 2015; Tóth et al. 2016; Reimann et al. 2017; Navazas et al. 2019). Mid-2018, 1355 out of 6808 French polluted sites (19.9%) display an As contamination, including 806 sites for soils and 317 ones for soils and groundwater (Antoni et al. 2019). More than 600 As-contaminated sites require remediation in the USA (da Silva et al. 2018a, 2019a). Reducing As accumulation in rice is a top priority in the management of Chinese contaminated soils (Zhao 2020).

Background values for total soil As (in mg kg^−1^) usually range from 0.1 to 67 with an average value around 5 (Singh et al. 2015; da Silva et al. 2018a), e.g. frequent French values: 1–25 and geochemical outliers: 30–60, median and mean values for French topsoils: 12 and 18 (Baize 2016). French topsoils exceeding potential guideline values (45–50 mg As kg^−1^) occur in localised hot-spots primarily attributed to geology and mineralisation, mining, pesticides, and some anthropogenic activities (Marchant et al. 2017). Mean, median, minimum, maximum, and outlier values in English topsoils are respectively (mg As kg^−1^): 16, 14.1, < 0.5, 1008, and 555–15,100 (Ander et al. 2013).

Major bioavailable As forms are arsenates (As(V), i.e. H_2_AsO_4_^−^ and HAsO_4_^2−^) and arsenites (As(III), i.e. H_3_AsO_3_, and H_2_AsO_3_^−^). Arsenical species depend on soil types, pH and their redox status, their toxicity ranking as As(III) > As(V) > organic forms: monomethylarsonic acid (MMA) > dimethylarsinic acid (DMA) (Jain and Ali 2000). Inorganic As forms react with soil Al/Fe/Mn oxides, Ca/Mg carbonates, and clay minerals whereas dissolved organic matter can promote As desorption (Kumpiene et al. 2019). Arsenical species display contrasting properties for their sorption to Fe/Mn-containing minerals, depending on soil pH and other soil factors (Vega et al. 2017; Kumpiene et al. 2019). As(V) generally predominates in aerobic conditions, while As(III) prevails under anaerobic conditions being more (bio)available than As(V). Various organic arsenicals are reported (Singh et al. 2015). Soil flooding and aerobic-anaerobic transitions affect As speciation, sorption, and bioavailability in relation to the soil redox status and potential releases due to Fe oxyhydroxide dissolution (Li et al. 2019b; Zhao 2020). One option to reduce As exposure in contaminated soils and water is the use of Fe/Mn-based minerals and their derivatives in line with As speciation. However, bioremediation options for As-contaminated soils must generally address both metal(loid)s and xenobiotics, accounting for benefits and limits, including energy and C balance (Plewniak et al. 2018; Gonzalez-Martinez et al. 2019).

Maximum permitted concentrations are in force or guideline values proposed in several countries according to the use (e.g. for drinking water and soil, WHO 10 µg As L^−1^ and US EPA 24 mg As kg^−1^, respectively), but this is hampered by the bioavailable As fraction and the variability of soil types. Remediation costs, time frame, water and soil volumes to treat with physico-chemical technologies, and by-products/secondary contamination to manage are frequently not financially and technically sustainable. This leads most countries to adopt a risk-based management system to manage/remediate polluted sites and soils, e.g. France (Info Terre 2017), UK (Jiang et al. 2015). This review aims to inventory the current knowledge on the interactions between As, microbes and plants, supporting the development of promising methods based on microbiological processes or phytotechnologies that could therefore be useful to reduce the harmful effects on human health due to As contamination of soils and groundwater.

## From microbial genomics to metagenomics

Throughout geological periods, microorganisms have occupied multiple ecological niches, including those whose physico-chemical conditions are deemed to be extreme. The diversity of their metabolic activities is pivotal in biogeochemical cycles, which can have a deep impact on water quality and soil productivity (Madsen 2011). In addition, they represent a huge gene reservoir, many of which are still of unknown function and which could present a strong potential for developing biotechnological applications (Yang and Ding 2014, Krüger et al. 2018).

The rise of molecular biology and the considerable advances in DNA sequencing methods have contributed to the emergence of genomics, whose methods aim to study the organisation and activity of living organisms based on the understanding of their genome (Bertin et al. 2015, Land et al. 2015). However, data on microbial diversity within ecosystems provided by conventional molecular methods have revealed that a large majority of microorganisms belong to taxa for which no representative has been isolated yet in pure culture (Rashid and Stingl 2015). Indeed, the culture of a majority of them can be extremely tedious and, therefore, accessing their genome and their metabolic potential could be facilitated by the use of environmental genomics methods. For example, the genome of a new uncultured betaproteobacterial species was assembled from metagenomic data obtained from a polymetallic mine. The physiology of this strain, i.e. ‘*Candidatus* Gallionella acididurans’, was investigated, in particular regarding Fe metabolism (Kadnikov et al. 2016). Likewise, the genome of a *Ferrovum* bacterial strain, able to oxidise Fe, was rebuilt using a mixed culture made from samples taken in a mining water treatment plant (Ullrich et al. 2016), and both metagenomic and metatranscriptomic data (Plewniak et al. 2020).

Combined with genome bioinformatic analysis, molecular techniques have proven to be valuable tools for deciphering genomic data (Vallenet et al. 2017, Machado et al. 2017). Nevertheless, cultural approaches are still required in microbiology to broaden our knowledge of the microorganism physiology for exploiting their properties in bioremediation strategies (Overmann et al. 2017). Such approaches represent a significant challenge because microorganisms isolated from various environments can grow extremely slowly and may require specific nutrients and growth conditions. Consequently, the genome characterisation of an organism can be used to try to identify its metabolic characteristics and use them to attempt to cultivate it (Garza and Dutilh 2015). The isolation of *Leptospirillum ferrodiazotrophum*, an acidophilic Fe-oxidising bacterium is an example (Tyson et al. 2005). Similarly, from environmental DNA sequencing, non-cultured ‘*Candidatus* Desulforudis audaxviator’ was described as virtually the only species identified in a gold mine (Chivian et al. 2008), until another strain with a highly similar genome was isolated from a deep aquifer in Siberia and grown in laboratory conditions (Karnachuk et al. 2019).

**Fig. 1:**
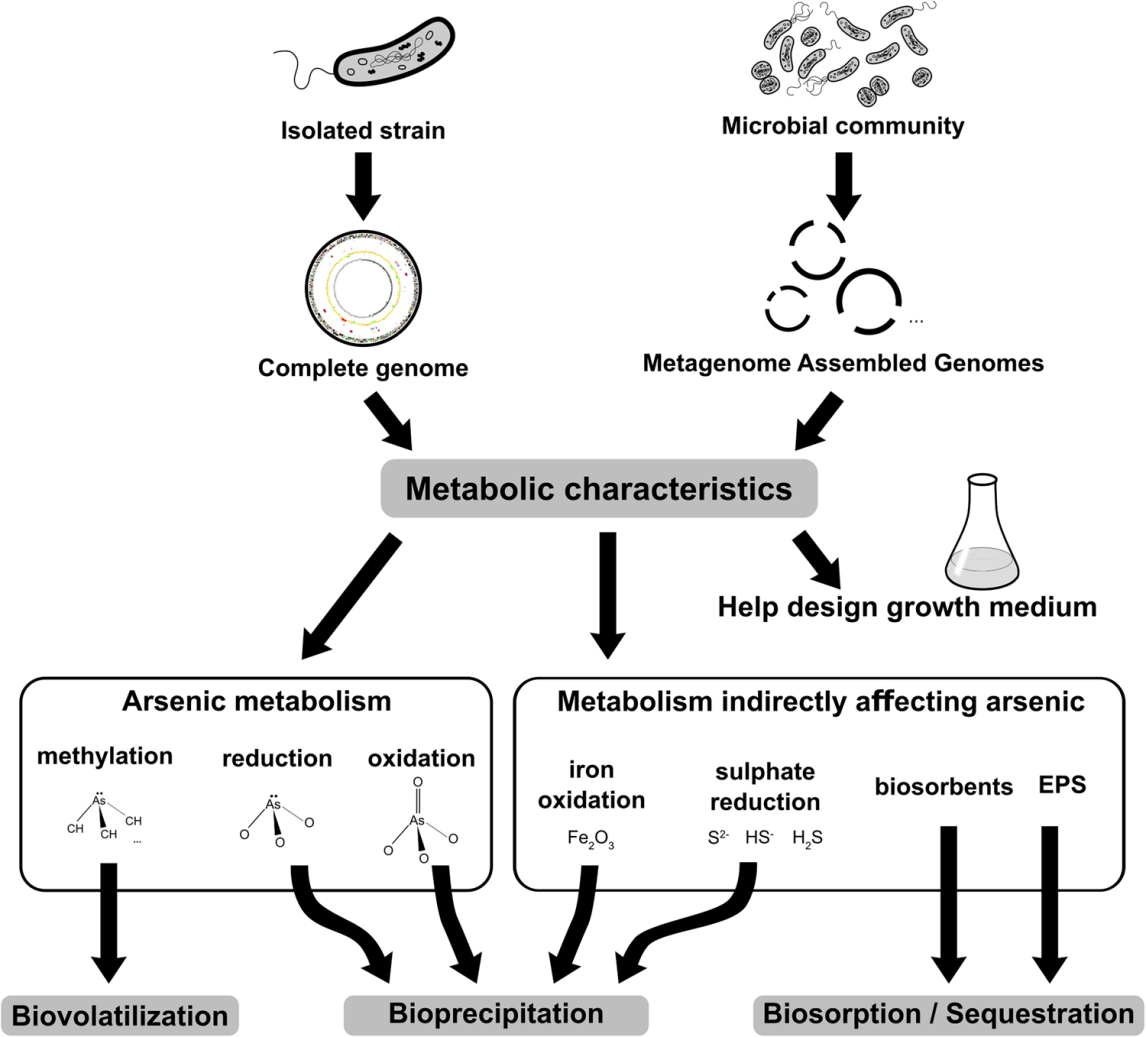
Genomic studies applied to isolated microorganisms and microbial communities can provide information about their metabolic capacities. Predictions of trophic and energy metabolism may thus help design or improve an efficient growing medium. Other metabolic activities involving arsenic (methylation, reduction, and oxidation) or whose products can interact with arsenic may be leveraged for As removal by either volatilisation, precipitation, or adsorption

The genome of several microorganisms metabolising As has been characterised from various ecosystems, as well as the genes involved in the various aspects of this metabolism (Fig. 1, Andres and Bertin 2016, Ben Fekih et al. 2018). The first described bacterium is *Herminiimonas arsenicoxydans*, a β-proteobacterium isolated from an industrial water treatment plant. It is resistant to high As concentrations and able to oxidise As(III) to As(V) (Muller et al. 2007). Based on functional genomics, a biphasic response to the As presence occurs: *H. arsenicoxydans* firstly activates resistance mechanisms partly based on efflux pumps; then the metabolic processes driving to the As(III) oxidation are triggered (Cleiss-Arnold et al. 2010, Koechler et al. 2010). Based on electron microscopy, this strain synthesises an exopolysaccharide (extracellular polymeric substance: EPS) able to sequester As in its matrix (Muller et al. 2007). The strain *Thiomonas* sp. 3As, isolated from an abandoned mine in southern France, also produces significant EPS amounts when exposed to arsenite; it would be therefore a relevant candidate for developing bioremediation processes based on biofilm-based bioreactors (Arsène-Ploetze et al. 2010). Unlike the previous two, a *Rhizobium* strain isolated from a gold mine in Australia carries the genes involved in the resistance and detoxification of As on a plasmid. Such a genetic tool could be interesting from a phytoremediation perspective by transferring the As detoxification capacity to related plant-associated bacteria (Andres et al. 2013). Finally, we can mention the genome of two hyper-As(III)-tolerant strains able to oxidise arsenite: *Halomonas* A3H3 and *Pseudomonas xanthomarina* S11, respectively isolated from the Mediterranean Sea-contaminated sediments (Koechler et al. 2013) and a French old gold mine (Koechler et al. 2015).

High-throughput sequencing techniques and the development of assembly software are continuously improved and facilitate to determine the genomic sequences of non-cultured microorganisms by direct sequencing of environmental DNA extracted from complex microbial communities (Fig. 1). Even though some problems remain concerning notably sampling, collating, and annotating (Thomas et al. 2012, Teeling and Glöckner 2012), in 2018 the Genomes Online database contained nearly 40,000 analysed metagenomes (Mukherjee et al. 2018). This number should further increase accounting for gigantic projects like the Earth Microbiome Project. Nearly 500,000 genomes would indeed be used to build a global genetic atlas of microbial communities (Gilbert et al. 2010; Thompson et al. 2017; Danko et al. 2019).

Currently, environmental descriptive and functional genomics can provide a comprehensive overview of microbial communities extending studies focusing on specific organisms. Such a global vision of both community structure and functioning would improve our understanding of natural remediation processes and help to find candidate species suitable for designing bioprocesses. In line, ecological questions related to the diversity and dynamics of microbial populations can be addressed using high-throughput sequencing methods. For example, bacterial, archaeal and fungal communities in rice paddy soils were inventoried by such approach, and shown to be strongly affected by irrigation with waters contaminated by metals such as Cu, Pb, and Zn (Wang et al. 2018b). Environmental DNA and RNA sequencing was also successfully used to analyse the response of bacteria belonging to a new deltaproteobacterial order, ‘*Candidatus* acidulodesulfobacterales’. Based on metabolic pathways reconstructed from metagenome-assembled genomes (MAG) and gene expression profiles, these microorganisms would be facultative anaerobic autotrophs possibly involved in Fe cycling (Tan et al. 2019).

Similarly, environmental genomics have been used on As-contaminated ecosystems (Andres and Bertin 2016; Huang et al. 2016). For example, seven microbial genomes present in an acid mine drainage (AMD) (Carnoulès, France) were almost completely reconstructed. The combination of the metabolic activities of the corresponding microorganisms, in particular the oxidation of arsenite and its co-precipitation with Fe and S, leads to a natural attenuation process, which greatly reduces the As concentration along the stream (Bertin et al. 2011). Recently, a metatranscriptomic study of Carnoulès AMD sediment samples shed light on how food webs may affect the structure and activities of microbial communities in such environments. In particular C:N and N:P ratios, influenced by the presence of metazoa and the riparian vegetation, may be pivotal, along with predation patterns, in shaping microbial communities (Plewniak et al. 2021). Another study has compared sediments extracted from two ports in the Mediterranean Sea. The specific sequences belonging to bacteria metabolising S match with both the biotic reduction of sulfates and the abiotic production of thioarsenical compounds. These elements being highly soluble, this likely explains why the most contaminated site has higher As mobility (Plewniak et al. 2013). Moreover, 27 genomes of Micrarcheota and 12 Parvarchaeota were assembled from 12 metagenomes from the Richmond mine, California. These organisms could participate in C and N cycles by degrading organic matter and be key players in Fe oxidation (Chen et al. 2017). Therefore, many microbial communities and their hosted organisms metabolising Fe, S, and As could be candidates for the development of novel bioremediation processes. In this regard, sulfate-reducing bacteria resistant to metal(loid)s and acidic conditions were used to remove As from an AMD (Serrano and Leiva 2017).

## Arsenic microbial metabolisms and bioremediation

Conventional technologies for As removal from As-rich waters mainly include physico-chemical treatments like alum, Fe and Mn precipitation, enhanced lime softening, ion exchange, electro dialysis, reverse osmosis, coagulation/filtration, and adsorption (Ng et al. 2004; Nicomel et al. 2016). For reducing management costs and enhancing the water treatment capacity, several adsorption technologies have been developed (Mohan and Pittman 2007; Nicomel et al. 2016). Considering that these technologies are very effective in removing As(V), a pre-oxidation step allowing the conversion of As(III) to As(V) is often required. For this purpose, strong chemical oxidising agents are commonly utilised (Katsoyiannis et al. 2002; Simeonova et al. 2005).

Over the last years, the use of biological processes for As removal has been widely investigated and a large number of potential applications were proposed to remediate As-contaminated ecosystems due to their environmental compatibility and cost-effectiveness (Kruger et al. 2013; Plewniak et al. 2018; Sher and Rehman 2019; Upadhyay et al. 2018; Wang and Zhao 2009). Among the bacterial As-remediation processes, biosynthesis of adsorbent materials, biovolatilisation, bioprecipitation and biosorption are mostly applied (Fig. 1, Fazi et al. 2016a). The occurrence of sulfides and biogenic iron oxides in groundwater facilitates As bioprecipitation resulting in a low As concentration (Omoregie et al. 2013). Many bacteria, indeed, are able to reduce As-, Fe-, and Mn-bearing minerals and promote As sorption onto freshly formed hydrous ferric oxide (HFO) (Katsoyiannis and Zouboulis 2004; Omoregie et al. 2013). Many microorganisms may also produce adsorbent materials, such as FeOOH nanoparticles within extracellular polymeric substance (EPS) hydrogel (Fe-EPS), used to treat As-rich drinking water (Casentini et al. 2015; Mandal et al. 2006). In addition, biosorption processes could be applied for removing metal(loid)s and other elements from diluted aqueous solutions even though few studies reported its application in drinking water treatment (Mohan and Pittman 2007; Hasan et al. 2010; Prasad et al. 2013).

Although with a limited impact on aquifer contamination, biovolatilisation may contribute to the As removal from soil and water (Jakob et al. 2010; Lloyd 2010; Liu et al. 2011). Arsenic methylation is considered a key player of the As cycle on Earth (Bhattacharjee and Rosen 2007). Volatile arsenic is formed through a consecutive transformation of inorganic As to methylated species (Rahman et al. 2014). In spite of this process being widely investigated, its exploitation for bioremediation purposes is still limited (Zhang et al. 2015a; Wang and Zhao 2009). Indeed, although many microbes may aerobically or anaerobically perform the methylation of As species, low rates of biological As volatilisation are reported in soil (< 10% of total As content) (Liu et al. 2011). The first described microorganism able to convert As(V) to volatile methylarsines is *Methanobacterium bryantii* (McBride and Wolfe 1971). *Achromobacter* sp. and *Enterobacter* sp. have the capability to convert As(V) to mono- and di-methylarsine, while *Aeromonas* sp. and *Nocardia* sp transform this element in mono-, di-, and trimethylarsine (Cullen and Reimer 1989). Although the volatilisation is considered a detoxification process, it produces highly toxic species whose availability in soils and groundwater can represent a serious threat (Bentley and Chasteen 2002; Wang and Mulligan 2006).

### Arsenic oxidising microorganisms

Among the bioprocesses involved in the regulation of As biogeochemical cycle in aquifers, the capability of microorganisms to transform As through oxidation–reduction reactions are of great interest in As bioremediation applications. In recent years, the As(III) oxidation mediated by microorganisms has assumed increasing importance as a precursor step of commonly used iron-based treatment methods (Crognale et al. 2017; Fazi et al. 2016a). Usually, chemical oxidising reagents (e.g. chlorine, potassium permanganate, manganese oxide, hydrogen peroxide, and ozone) are added to the water (Driehaus et al. 1995; Kim and Nriagu 2000). However, this chemical pre-oxidation may cause secondary problems due to the occurrence of residuals or by-products formation, and a significant increase in operational costs (Katsoyiannis and Zouboulis 2004). To circumvent these limitations, the microbiological As(III) oxidation has been proposed as an eco-friendly alternative to conventional chemical pre-treatment methods (Bahar et al. 2013). Several As(III)-oxidising microorganisms have been recovered in various As-rich environments including geothermal sites, soils, sediments, mine, arsenical pesticides and smelter-impacted sites (Engel et al. 2013; Fazi et al. 2016b; Heinrich-Salmeron et al. 2011; Lami et al. 2013; Paul et al. 2018; Quéméneur et al. 2008, 2010; Satyapal et al. 2018; Sultana et al. 2012; Thul et al. 2019). The As(III) oxidation is a detoxification process in heterotrophic bacteria (Bahar et al. 2012; Muller et al. 2003; Vanden Hoven and Santini 2004), or an energetic metabolism in chemolithoautotrophic microorganisms, such as *Rhizobium* NT-26 and *T. arsenivorans* (Battaglia-Brunet et al. 2006; Garcia-Dominguez et al. 2008; Hoeft et al. 2007; Santini et al. 2000). The capability of microorganisms to anaerobically oxidize As(III) in combination with nitrate respiration or anoxygenic photosynthesis is reported in several studies (Cui et al. 2018; Hoeft et al. 2007; Kulp et al. 2008; Ospino et al. 2019; Zargar et al. 2012; Zhang et al. 2015b, 2017). Over the last years, lab-scale experiments have been performed on immobilised bacteria, biofilms, and planktonic cells to better elucidate the potential of the aerobic biological As(III) oxidation process in water treatment (Battaglia-Brunet et al. 2002; Dastidar and Wang 2012; Ito et al. 2012; Michel et al. 2007; Michon et al. 2010). The capability to oxidise As(III) is reported in several bacterial strains, e.g. *Aliihoeflea* sp. 2WW, *Bacillus* spp., *Bosea* sp. AS-1, *Delftia* spp. BAs29, *Ensifer adhaerens*, *Micrococcus* sp., *Pseudomonas chengduensis*, and *T. arsenivorans* (Biswas and Sarkar 2019; Biswas et al. 2019; Corsini et al. 2014; Ito et al. 2012; Jebelli et al. 2018; Lu et al. 2018; Roychowdhury et al. 2018; Wan et al. 2010).

A fixed bed up-flow filtration unit allowing for the simultaneous biotic oxidation and removal of As(III) and Fe(II)/Mn(II) has been developed and tested in several studies (Hassan et al. 2009; Katsoyiannis and Zouboulis 2004; Katsoyiannis et al. 2004; Tani et al. 2004). Few investigations have combined biological As(III) oxidation with the use of activated alumina and metallic Fe adsorbents to remove As (Ike et al. 2008; Wan et al. 2010). The use of a polarised electrode as terminal electron acceptor for the bioelectrochemical As(III) oxidation has been recently investigated (Pous et al. 2015; Nguyen et al. 2017). Additionally, the cathodic electroactivity of a new chemolithoautotrophic arsenite oxidising bacterium, *Ancylobacter* Ts-1 has been proved suggesting the potential application of bioelectrochemical process in bioremediation of natural and bio-engineered environments (Anguita et al. 2018).

As(III) oxidising biofilms can play a key role in the design of simple passive bio-processes (Battaglia-Brunet et al. 2005). Initially, this approach has been investigated by using mixed microbial communities or populations recovered from As-rich extreme environments. For example, an autotrophic As(III) oxidising bacterial population, named CAsO1, has been tested at lab-scale in a fixed bed column reactor revealing a high capability in oxidising As(III) at a rate of 166 mg L^−1^ h^−1^ (Battaglia-Brunet et al. 2002, 2005). Due to the formation of an EPS matrix, the biofilm may act as a physical barrier for several particles, cations, anions and apolar compounds occurring in the water (Flemming and Wingender 2010; Michel et al. 2007). The application of As(III) oxidising biofilms in combination with Fe- and Fe-/Mn-oxidation processes may increase the efficiency of simultaneous As-, Fe-, and Mn- removal from groundwater (Casiot et al. 2006; Hassan et al. 2009; Katsoyiannis and Zouboulis 2004; Katsoyiannis et al. 2004, 2007; Tani et al. 2004). Moreover, the As(III)-oxidising biofilters have high potentialities in the treatment of As-contaminated groundwater (Crognale et al. 2019; Gude et al. 2018; Li et al. 2016; Yang et al. 2014). Up to 150 mg As L^−1^ ( ~ 98.2% of total As), 1.5 mg Fe L^−1^ and 1.2 mg Mn L^−1^ have been simultaneously removed from groundwater within 180 days by using quartz sand biofilter (Yang et al. 2014). Li et al. (2016) reported the capability of a lab-scale biofilter, inoculated with an As(III)-oxidising population to oxidise 1.1 mg As(III) L^−1^ within 10 min. Gude et al. (2018) have tested the As(III) oxidising potentialities of a biofilter started up with a native As-rich groundwater microbial community showing the importance of initial acclimation to As(III)-rich groundwater. Indeed, up to 98% of 0.1 mg L^−1^ of As(III) has been oxidised in 38 days by using a not-acclimated biofilter and within three weeks with a biofilter previously exposed to As-contaminated groundwater (Gude et al. 2018). Crognale et al. (2019) have tested the biofilter potentialities using autochthonous As-rich groundwater microbial communities under experimental conditions mimicking those used in household-scale treatment system (Casentini et al. 2016). This study evidenced a high oxidation efficiency (up to 90% of 0.1 mg As(III) L^−1^ in 3 h) in a biofilter filled with coarse sand.

The use of combined As(III) and Fe(II) bio-oxidising activities was developed for treating a neutral pH surface water containing 5 to 12 mg Fe L^−1^ and 0.2 to 2.0 mg As L^−1^ - with 50 to 100% As(III) - in an entirely passive system at the Loperec site (Finistère, France) (Battaglia-Brunet et al. 2006). Bacteria developed as a biofilm composed of a complex community, on pozzolana support, and promoted the precipitation of Fe hydroxides (ferrihydrite) as an efficient adsorbent for As(V) resulting from bacterial As(III) oxidation. This process implemented at real scale since 2017 is treating an average 15 m^3^ h^−1^ flux of water flowing from an exploration gallery. The residence time optimised at the laboratory could be decreased to 0.5 h without affecting the treatment efficiency, the total As concentration being reduced below the 100 µg L^−1^ limit for discharge in the environment.

### Bioreduction mechanisms for As bio-precipitation

In low-pH conditions, characteristic of AMD, efficient Fe precipitation can be problematic. In other polluted waters, Fe concentration in the water to be treated is too low for the efficient co-precipitation of As with Fe. An alternative to oxidising processes can be the bio-precipitation of As sulfides with the help of bacteria able to perform the dissimilatory reduction of sulfate, sulfate-reducing bacteria (SRB), and bacteria able to reduce As(V) into As(III), either through dissimilatory As(V) reduction or As(V) reduction linked to the As resistance system. Arsenate respiration is based on the activity of the arsenate respiratory reductase (ARR, Afkar et al. 2003). This enzyme is a periplasmic dimethyl sulfoxide (DMSO)-type reductase that reduces As(V) to As(III) (Saltikov and Newman 2003). Arsenic, in the As(III) state, precipitates with sulfide to form the insoluble yellow amorphous orpiment As_2_S_3_. Eary (1992) was the first to report solubility data for amorphous As_2_S_3_, which suggested the following two equilibria:

1 $${\mathrm{H}}_{3}{\mathrm{AsO}}_{3} + 1.5 {\mathrm{H}}_{2}\mathrm{S }= 0.5\mathrm{ A}{\mathrm{s}}_{2}{\mathrm{S}}_{3} (\mathrm{am}) + 3 {\mathrm{H}}_{2}\mathrm{O}\mathrm{\ log }K = -11.9$$

2 $$1.5\mathrm{ A}{\mathrm{s}}_{2}{\mathrm{S}}_{3} (\mathrm{am}) + 1.5 {\mathrm{H}}_{2}\mathrm{S }= {\mathrm{H}}_{2}{\mathrm{As}}_{3}{{\mathrm{S}}_{6}}^{-} + {\mathrm{H}}^{+}\mathrm{\ log }K = -5$$

When sulfide concentration exceed 1 mg L^−1^, soluble complex thioarsenate species would form at circa neutral and higher pH (Smieja and Wilkin 2003).

The first characterised sulfate-reducing organism able to use As(V) as terminal electron acceptor was isolated from surface sediments of the Upper Mystic Lake, Massachusetts, USA (Newman et al. 1997a, b). This bacterium described as *Desulfosporosinus auripigmenti* (Stackebrandt et al. 2003) is a freshwater, gram-positive, non-motile, strictly anaerobic chemoorganotroph. It oxidises H_2_, lactate, pyruvate, butyrate, ethanol, glycerol, and malate for its growth concomitantly with the reduction of either sulfate or arsenate. Macy et al. (2000) worked with two SRB able to reduce both As(V) and SO_4_ concomitantly. One of them, *Desulfomicrobium* Ben-RB, used As(V) as terminal electron acceptor. The second SRB, *Desulfovibrio* Ben-RA, reduced As(V) through an As resistance system. Although *Desulfovibrio* Ben-RA did not perform dissimilatory As(V) reduction, it was able to promote As_2_S_3_ precipitation. Both *D. auripigmenti*, *Desulfomicrobium* Ben-RB and *Desulfovibrio* Ben-RA were grown in near-neutral pH conditions.

Another SRB related to *D. auripigmenti* was isolated from sediments of the polluted Onondaga Lake (Syracuse, NY, USA). This As(V) respiring isolate, named strain Y5, can utilise aromatic substrates (Liu et al. 2004). Both *D. auripigmenti* and strain Y5 are spore-forming rods. The lack of motility differentiates *D. auripigmenti* from the other *Desulfosporosinus* strains (*D. orientis, D. meridiei* and strain Y5). In this group, only *D. auripigmenti* and strain Y5 are known to reduce As(V). Demergasso et al. (2007) obtained enrichments and isolates from boron deposits in Andean salt flats. Two strains isolated, CC-1 and Asc-3 grew using SO_4_ or As(V) as electron acceptors. The nearest phylogenetic relatives (based on 16S rRNA sequences) of CC-1 and Asc-3 are *Pseudomonas* sp. PHLL and *Enterobacter* sp. BL2, respectively.

**Fig. 2:**
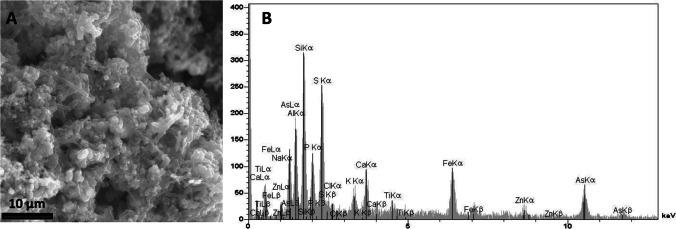
SEM observation (**A**) of biofilm embedded in bioprecipitates in a sulfate-reducing column bioreactor fed with a 100-mg As L^−1^ solution (Battaglia-Brunet et al. 2012) and (**B**) corresponding EDS spectrum analysis. EDWARDS Auto 306 apparatus (EMS, Hatfield, PA, USA), then observed using a JSM 6100 Scanning Electron Microscope (JEOL, Tokyo, Japan) coupled to an X-ray Energy Dispersive Spectrometer KEVEX Quantum (Thermo Electron Corp., Dreieich, Germany)

Bio-precipitation of As sulfide from synthetic acid solutions was performed in continuously fed laboratory column bioreactor (Battaglia-Brunet et al. 2012). In this system, the acid (pH 2.7 to 5) feeding solution contained 100 mg As(V) L^−1^, and glycerol or H_2_ were provided as energy sources. Bacteria embedded in As- and S-rich mineral phases were observed at the surface of the bioreactor filling material (Fig. 2). The removal rate reached 2.5 mg As L^−1^ h^−1^. The diverse bacterial community developed in the bioreactor included *Desulfosporosinus*-like sulfate-reducing bacteria and fermenting ones. The retrieved *arr*A sequences were 100% identical to that of the As(V)-respiring SRB strain *Desulfosporosinus* Y5 (Liu et al. 2004); this suggested that a *Desulfosporosinus*-related bacteria contributed to As(V) reduction via a dissimilatory mechanism.

With a real As-rich AMD water containing 90 mg As L^−1^ (Carnoules site, Gard, France), growth of SRB inducing precipitation of the As sulfides orpiment and realgar, together with ZnS nanoparticles, has been proven in batch experiments (Le Pape et al. 2017). This bioprocess was then tested at laboratory scale in continuous feeding conditions (Battaglia-Brunet et al. 2021) and is currently up-scaled to be tested on site. Up to now, As removal in SRB systems was reported at real scale on a few sites, but with mine waters and effluents less acidic than that of the Carnoules site. One is located at the Wood Cadillac mine, Northwestern Quebec, which features sulfide poor but As rich, tailings, laid down between 1939 and 1949 (Tassé et al. 2003). A biofilter (50 m × 57 m, 1 m thick, vertical flow and residence time of 25 h) was implemented, with wood chips as low-rate delivered energy source, to treat mine water at pH 6–7 with relatively low As concentrations (0.1 to 1.2 mg L^−1^). Another real scale application has been implemented near the city of Trail in British Columbia, Canada (Al et al. 2011). Anaerobic biofilters filled with a mixture of limestone, quartz sand and biosolids, a by-product of the pulp and paper industry, are treating effluent water from smelting operations at pH 5.9 containing around 50 mg As L^−1^. The size of both bioreactors in series are 18 × 30 m and 18 × 25 m, and the residence time of the water in these two systems is 720 and 600 h, respectively.

## Phytoremediation of As-contaminated soils

Phosphate transporters (Pht) mediate root uptake of As(V), which competes with phosphates (Farooq et al. 2016; Kofronova et al. 2018, Vromman et al. 2018, Zhao and Wang 2020). In *Pteris vittata* (As hyperaccumulator) three genes would collaborate, i.e. *PvPht1;3*, a phosphate (P) transporter gene; *PvACR2*, an As(V) reductase gene; and *PvACR3*, an As(III) transport gene, in sensitive As(V) absorption, constitutive As(V) reduction, and subsequent As(III) transportation (Wei et al. 2021). Several elements, i.e. Si, Se, Fe, P, and Mo, can challenge As uptake by roots (Mu et al. 2019). As(V) taken up is mostly reduced to As(III) through arsenate reductases in higher plant tissues (Triptahti et al. 2012; Kofronova et al. 2018). The protein high arsenic concentration 1 (HAC1) would drive As(V) reductase activity in the outer root layer (epidermis) and the inner one adjacent to the xylem (pericycle). Nodulin 26-like intrinsic protein (NIPs) aquaporine channel transports As(III) in the plants; the OsLsi1/NIP2;1 transporter, mediating transport of silicic acid into roots, is expressed in the zone of Casparian strips at plasma membrane and contributes to As(III) intake in rice and maize. The As methylated forms display generally a lower uptake rate than that of the inorganic ones. Monomethylarsonic acid (MMA) and dimethylarsinic acid (DMA) are detected in plant parts (Kofronova et al. 2018). Arabidopsis plants with mutated inositol transporter genes (i.e. AtINT2, AtINT4) exhibit lower As(III) concentrations in the phloem. As(III) binding to sulfhydryl groups can affect the function of several proteins; it can be chelated by phytochelatins (PCs) and then transported and sequestered in vacuoles (Zhao et al. 2010; Triptahti et al. 2012; Zhao and Wang 2020; Wei et al. 2021). Arsenic forms differ between root tissues. The translocation of As species from roots to shoots depends on plant species: As(III) predominates in the xylem sap of tomato, cucumber, rice, and the fern *Pteris vittata*; larger As(V) amounts occur in Indian mustard, wheat, and barley. Arsenic excess can be phytotoxic: it decreases plant growth, alters inorganic nutrition, impairs the plant water status, generates oxidative stress and may be a nitrosative one, restrains photosynthesis, and changes the hormonal content. This leads to physiological disruptions and finally the plants die (Farooq et al. 2016). As(III) inactivates many enzymes by disorganising their structure and damages the metabolism by impeding protein–protein interactions (Navazas et al. 2019). This impacts many cellular metabolic key processes, e.g. glucose uptake, glutathione production, and fatty acid metabolism. For reviews on plant responses to As excess, see, e.g. Clemens and Ma (2016), Kofronova et al. (2018), Zhang et al. (2018), Zhao (2020), and Wei et al. (2021).

Obvious ways to alleviate pollutant linkages due to As excess are to avoid sources leading to As contamination and exposure, to select plant species and associated microorganisms for minimising As concentration in edible plant parts, and to reduce As in exposure pathways (Kofronova et al. 2018; Zhao 2020). Physico-chemical technologies, e.g. oxidation, coagulation-flocculation, soil washing, adsorption, ion exchange, electrokinetics and membrane technologies, are reviewed elsewhere (Singh et al. 2015; Kumpiene et al. 2019). Development of efficient, less-invasive remediation (phyto)technologies is crucial for soils and water contaminated with both metal(loid)s and xenobiotics, notably to phytostabilise/exclude or phytoextract As, and large volume/area. Several shortcomings accompany As phytoextraction. One is the number of successive crops needed to extract bioavailable soil As (the remediation time should not be estimated on the basis of total soil As). The phytoextraction option is also limited to the surface soil (~ 1 m). For all phytomanagement options, heatwaves, drought, late frost, pests, etc. and hydric conditions may hinder the plant development. Climate change must be considered, including storms to prevent flooding and erosion.

Arsenic is tolerated and accumulated in roots, with low translocation in shoots, by many plant species, so-called excluders. Some others retain As in their roots but also display high shoot As concentration, i.e. (hyper)accumulators. Use of these plant phenotypes leads to various phytotechnologies and phytomanagement options, from sequestration of labile As pools in the root zone (exclusion, phytostabilisation) to harvest of As accumulated in plant parts (phytoextraction) (Hettick et al. 2015; Wang et al. 2017). *Pteris vittata* (Chinese brake fern) is a notorious As hyperaccumulator, its fronds being able to amass up to 23 g As kg^−1^ (da Silva et al. 2018a). These phytoremediation options influence physico-chemical and biological soil properties underlying ecosystem services, including gain in biodiversity and resilience to metal(loid) excess and climate changes (Mench et al. 2003a, b, 2010, 2014; Renella et al. 2008; Kidd et al. 2015).

Phytovolatilisation of arsenicals during phytomanagement is questionable. Dimethylchloroarsine (AsCl(CH_3_)_2_) and pentamethylarsine (As(CH_3_)_5_) were released by rabbitfoot grass (*Polypogon monspeliensis*) but not the more toxic organic As forms, i.e. arsine, MMA, DMA, and trimethylarsinic acid (TMA) (Ruppert et al. 2013). Phytovolatilisation of arsenicals from fronds of *P. vittata* grown in As-contaminated soil was claimed, but the method used is controversial and may just reflect evapotranspiration and As leaching from fronds (Sakakibara et al. 2010).

### Phytomanagement concept

Phytomanagement options (POs) for remediating contaminated land are a set of long-term, risk management phytotechnologies, involving plants and associated microorganisms, that promote a profitable crop production or other beneficial land uses (e.g. recreational park) and also lead gradually to the reduction of pollutant linkages due to contaminant excess (e.g. As) and a net gain in soil ecological functions underlying ecosystem services (Cundy et al. 2016). POs can be customised along contaminant linkages related to site/contaminant specificity and time frame, and can provide a wide range of environmental, economic and societal profits during and after the polluted land phytomanagement (Kidd et al. 2015). POs encompass the former phytoremediation options, which are long treatment time, but the phytomanagement concept overtakes them. One main purpose is to assist low-and medium-level polluted sites to return to productive usage, including either urban design, landscape architecture, or community gardens/parkland, in rural, urban, and suburban areas. The harvested biomass, produced by excluders or hyperaccumulators, is not a waste to dispose but a resource.

**Fig. 3:**
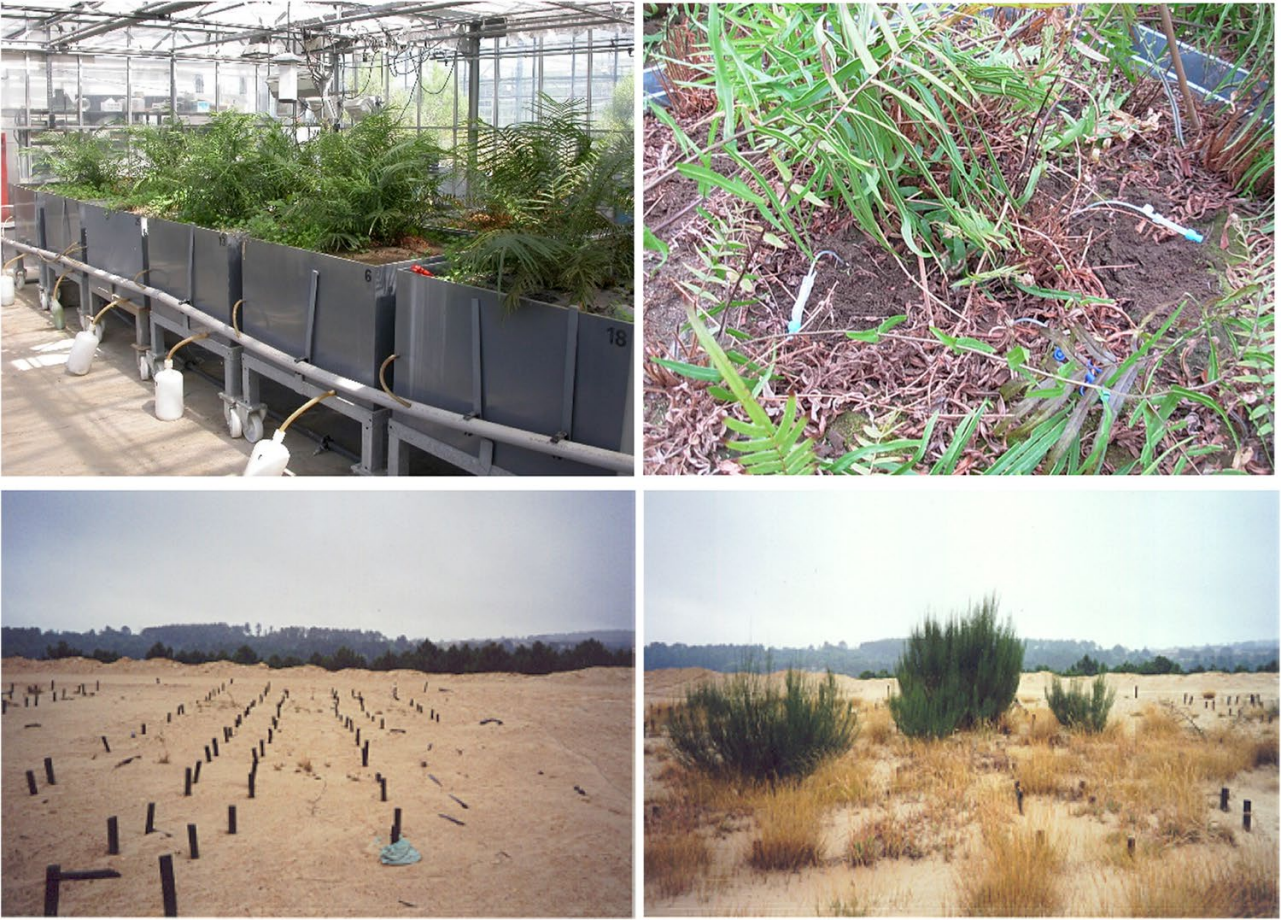
Clockwise: *Pteris vittata* growing on the As-contaminated Reppel soil placed in large mesocosms at the INRAE research center, Villenave d’Ornon, France (Phytorehab and Greenland EU projects); focus on passive samplers of soil pore water (Rhizon) inserted for monitoring changes in As exposure in the *P. vittata* rhizosphere; year 2 of the phytostabilisation field trial implemented at the Jales tailings, Portugal (EU Phytorehab project, FP5): (left) untreated topsoil; (right) topsoil amended with compost, coal fly ashes (beringite), and iron grit and colonised by As-tolerant populations of *Agrostis castellana*, *Holcus lanatus*, and *Cytisus striatus*. Photo © Dr. M. Mench (in collaboration with Pr. J. Vangronsveld, Hasselt Universiteit, Dr. P. Bleeker and Dr T. De Koe, Bleeker et al. 2002)

Where the marked lengthy time scales needed to achieve soil remediation, notably if based on total soil contaminants, has banned phytoremediation as a common technical option for urban polluted sites, phytomanagement, notably with either excluders or bioavailable contaminant stripping, is more considered by local and national authorities. (Aided) phytoextraction consists to (yearly) strip phytoavailable contaminants accumulate in harvestable plant parts (accounting for tissue concentration and biomass), eventually in combination with soil conditioners. It is mainly applicable to reduce pollutant linkages for large soil areas or water volume with low and medium exposure levels. However, phytoextraction using As-hyperaccumulators may be challenged, due to their specificity, at sites with multiple contaminants. Potential candidates for As-focused phytoremediation options are worldwide explored in hydroponics, pot experiments and field surveys (Table 1, Jiang et al. 2015). Only pot experiments with real As-contaminated soils (not spiked with salts), without dust exposure (for shoots collected on contaminated sites, dust trapped in stomatal chambers and wax cannot be simply washed) and giving information on plant growth and health (no necrotic plants), and thereafter confirm by field trials (accounting for climatic conditions and water supply), can validate such candidates (Table 1; Fig. 3). Among As-tolerant plant species, van der Ent et al. (2013) suggest 1000 mg As kg^−1^ DW in shoots as hyperaccumulation threshold criteria (excluding shoots contaminated by dust particles, notably in stomatal chambers), however shoot As removal (and bioavailable As stripping) is also a matter of biomass. Hyperaccumulators frequently have a low biomass production, and tolerance to either one, two, or rarely more elements, which limits the metal(loid) phytoextraction. In addition, their resilience to xenobiotic exposure at site with mixed pollution is poorly explored. In contrast, root compartments and excluders are useful to sequester bioavailable As in the root zone, but As phytostabilization can be only claimed when decreases in bioavailable soil As and As-focused pollutant linkages are demonstrated.

**Table 1:** Candidate plant species for As-focused phytoremediation in hydroponics, pot experiments, and field trials

Plant species	Exposure/growth period	Phenotype for As	Remediation option	Reference
Field experiments				
*Lolium* spp*.*, *Eschscholzia californica*	6 years	Excluders	Phytostabilisation	Pardo et al. (2018)
*Pityrogramma calomelanos*	136–269 mg As kg^−1^, 12 weeks	Hyperaccumulator	Phytoextraction	Jankong et al. (2007)
*Pityrogramma calomelanos*, *Pteris vittata*	324–909 mg As kg^−1^	Hyperaccumulators	Phytoextraction	Niazi et al. (2012)
*Dicranopteris linearis*, *Histiopteris incisa*, *Nephrolepis hirsutula*, *Pinus* sp., *Thysanolaena latifolia*, *Melastoma malabathricum*, *Pityrogramma calomelanos*, *Pteris vittata*	1091 mg As kg^−1^ mining site	Excluders; accumulators	Phytostabilisation; phytoextraction	Claveria et al. (2019)
*Agrostis castellana*, *Holcus lanatus*	1325 mg As kg^−1^, 4 years	Excluders	(Aided) phytostabilisation	Bleeker et al. (2002)
*Triticum aestivum* (*Pteris vittata*, *Phragmites australis*, *Vetiveria zizanioides*)	50 mg As kg^−1^, 2 years	Excluders	Phytostabilisation	Praveen et al. (2019)
*Lolium multiflorum*. var. *italicum*, *Secale cereale*, *Vicia villosa*, and *Trifolium pratense*	642 mg As kg^−1^, 3 months	Excluders	Phytostabilisation	Kim et al. (2018)
*Halogeton glomeratus*	3 mg kg^−1^, 1 year	Accumulator	Phytoextraction	Li et al. (2019a)
*Pteris vittata*	190 mg As kg^−1^, 2 years	Hyperaccumulator	Phytoextraction	Kertulis-Tartar et al. (2006)
*Pteris vittata*	26.7 and 129 mg As kg^−1^	Hyperaccumulator	Phytoextraction	da Silva et al. (2018a)
*Pteris vittata*		Hyperaccumulator	Phytoextraction	Gray et al. 2005; Shelmerdine et al. 2009 (cited in Jiang et al. 2015)
*Pteris vittata* intercropped with *Zea mays*	93 mg As kg^−1^	Hyperaccumulator + excluder	Phytoextraction; intercropping	Ma et al. (2018)
*Pteris ensiformis*, *Boehmeria nivea*, and 18 other species	125–6656 mg As kg^−1^		Phytoextraction; phytostabilisation	Pan et al. (2019)
*Oryza sativa*	72.7 mg As kg^−1^, 5 months	Excluder	Phytostabilisation/in situ immobilisation	Li et al. (2019b)
Watercourse/stream				
*Sagittaria montevidensis*			Rhizofiltration	Demarco et al. (2019)
20 macrophytes	9.7–13.6 mg As kg^−1^	Excluders	Phytostabilisation	Bonanno et al. (2018)
Outdoor lysimeters/vats				
*Pinus pinaster*	1325 mg As kg^−1^, 3 years	Excluders	Phytostabilisation	Mench et al. (2003a)
*Holcus lanatus*	1325 mg As kg^−1^, 3 years	Excluders	Phytostabilisation	Mench et al. (2003a)
*Pteris vittata*	113 mg As kg^−1^, 7 years	Hyperaccumulator	Phytoextraction	Mench et al. (2014)
Pot experiments				
*Dahlia pinnata*		Excluder	Phytostabilisation	Raza et al. (2019)
Aromatic plants for essential oils		Excluders	Phytoremediation	Pandey et al. (2019)
*Miscanthus sacchariflorus* A0104, *M. sinensis* C0424 and C0640	36–250 mg As kg^−1^ (spiked soils)	Excluders	Phytostabilisation	Jiang et al. (2018)
*Miscanthus x giganteus*	75–515 mg As kg^−1^ 2 years	Excluder	Phytostabilisation	Pidlisnyuk et al. (2019)
*Salix miyabeana* ‘SX67’	12 weeks CCA/PCDD/Fs	Excluder	Phytostabilisation	Fredette et al. (2019)
*Salix alba*, *Salix* sp.	Mine tailings	Excluders	Phytostabilisation	Vamerali et al. (2009)
*Salix viminalis*, *Salix purpurea*	Mine tailings	Excluders	Phytostabilisation	Bart et al. (2016)
*Populus*	Mine tailings	Excluder	Phytostabilisation	Vamerali et al. (2009)
*Populus nigra*	728 mg As kg^−1^, 28 days	Excluder	Phytostabilisation	Nandillon et al. (2019)
*Acer platanoides*	90 days	Excluder	Phytostabilisation	Budzynska et al. (2019b)
*Acer pseudoplatanus*	90 days	Excluder	Phytostabilisation	Budzynska et al. (2019b)
*Betula pendula*	90 days	Excluder	Phytostabilisation	Budzynska et al. (2019b)
*Quercus robur*	90 days	Excluder	Phytostabilisation	Budzynska et al. (2019b)
*Tilia cordata*	90 days	Excluder	Phytostabilisation	Budzynska et al. (2019b)
*Ulmus laevis*	90 days	Excluder	Phytostabilisation	Budzynska et al. (2019b)
*Jatropha curcas*	60–120 days, 18–1121 mg As kg^−1^	Excluder	Phytostabilisation	Alvarez-Mateos et al. (2019)
*Brassica juncea*	30 days	Indicator + K_2_HPO_4_ + PGPB	Aided phytoextraction	Franchi et al. (2019)
*Isatis cappadocica* cabbage (*Brassica oleracea* var. *sabauda*), broccoli (*B. oleracea* var. *italica*), cauliflower (*B. oleracea* var. *botrytis*)	145–6525/1825 mg As kg^−1^	Hyperaccumulators	Phytoextraction	Karimi et al. (2009)
*Zea mays*	30 days	Excluder + K_2_HPO_4_ + PGPB	Aided phytoextraction	Franchi et al. (2019)
*Helianthus annuus*	30 days	Excluder + K_2_HPO_4_ + PGPB	Aided phytoextraction	Franchi et al. (2019)
*Helianthus annuus*, *Lolium perenne*	15.9 g As kg^−1^	Excluders	Aided phytostabilisation	Vitkova et al. (2018)
*Lactuca sativa*	2 months		In situ immobilisation	Arco-Lázaro et al. (2018)
Barley; wheat	40–80 mg As kg^−1^ 4 months	Indicator; excluder	Phytoextraction; phytostabilisation	Gonzalez et al. (2019a)
*Brassica napus*	60 days	Excluder	Phytostabilisation	Gasco et al. (2019)
*Vetiveria zizanioides*	6 months	Excluder	Phytostabilisation	Mu et al. (2019)
*Oriza sativa*	0–100 mg kg^−1^	Excluder	Phytostabilisation	Irem et al. (2019)
*Arundo donax*	79.6 mg As kg^−1^, 9 months	Excluder	Co-planting with *P. vittata*	Zeng et al. (2019a)
*Arundo donax*, *Phragmites australis*	371 to 22,661 mg As kg^−1^	Excluders	Phytostabilisation	Castaldi et al. (2018)
*Morus alba*, *Broussonetia papyrifera*	41 mg As kg^−1^, 9 months	Excluders	Co-planting with *P. vittata*	Zeng et al. (2019a, b)
*Pteris cretica*	30 days, 200 mg As kg^−1^	Hyperaccumulator	Phytoextraction	Eze and Harvey (2018)
*Pteris vittata*		Hyperaccumulator	Phytoextraction	Yang et al. (2018)
*Pteris vittata*	251 mg As kg^−1^, 4 months	Hyperaccumulator	Phytoextraction	Wu et al. (2018)
*Pteris vittata*	65.8 mg As kg^−1^, 28 days	Hyperaccumulator	Phytoextraction	Wan et al. (2018)
*Pteris multifida*	0.5 mg As kg^−1^ (spiked soil), 3 months	Accumulator	Phytoextraction	Rahman et al. (2018)
*Holcus lanatus*	65.8 mg As kg^−1^, 28 days	Excluder	Phytostabilisation	Wan et al. (2018)
*Rosmarinus officinalis*	4–2738 mg As kg^−1^	Excluder	Phytostabilisation	Affholder et al. (2014)
*Polypogon monspeliensis*	250 mg As kg^−1^ (spiked soil)		Phytovolatilisation	Ruppert et al. (2013)
*Eupatorium cannabinum*	11 mg As L^−1^, 20 days	Excluder	Phytostabilisation	Gonzalez et al. (2019b)
Mesocosms/columns				
*Kosteletzkya pentacarpos*	75 mg As kg^−1^	Excluder	Rhizofiltration/phytostabilisation	Zhou et al. (2019)
*Tamarix gallica*		Excluder	Phytostabilisation	Sghaier et al. (2019)
*Phragmites australis*	8 months	Excluder	Phytoextraction	Cortes-Torres et al. (2019)
Constructed wetlands				
*Cyperus haspan*	85 µg L^−1^, 419 days	Indicator	Rhizofiltration	Corroto et al. (2019)
*Juncus effusus*	85 µg L^−1^, 419 days	Excluder	Rhizofiltration	Corroto et al. (2019)
*Colocasia esculenta*	0.48 mg L^−1^, 122 days	Excluder	Rhizofiltration	Vanlop et al. (2019)
Hydroponics				
*Salix atrocinerea*	18 mg As L^−1^, 30 days	Excluder	Phytostabilisation	Navazas et al. (2019)
*Salix* spp.		Excluder	Phytostabilisation	Purdy and Smart (2008)
*Salix purpurea* cv. ‘Fish Creek’	0–100 mg As/L (0–1335 µM)	Excluder	Phytostabilisation	Yanitch et al. (2017)
*Pteris vittata*	2 mM As(III) or As(V), 24 h	Hyperaccumulator	Phytoextraction	Yang et al. (2018)
*Pteris multifida*	21 µg L^−1^ As(III) (NaAsO_2_), 5 days; 33 µg L^−1^ As(III) 24 days	Hyperaccumulator	Phytoextraction	Rahman et al. (2018)
*Acer pseudoplatanus*	1 month, 1 mM As; 3 months,0.06–0.6 mM As(III), As(V), DMA	Excluder (sensitive)	Phytostabilisation	Budzynska et al. (2019a); Budzynska et al. (2018)
*Betula pendula*	1 month, 1 mM As	Excluder	Phytostabilisation	Budzynska et al. (2019b)
*Quercus robur*	1 month, 1 mM As	Excluder (sensitive)	Phytostabilisation	Budzynska et al. (2019b)
*Ulmus laevis*	1 month, 1 mM As	Excluder (sensitive)	Phytostabilisation	Budzynska et al. (2019b)
*Atriplex atacamensis*	2 weeks, 50 µM As(III) or As(V)	Excluder	Phytostabilisation/rhizofiltration	Vromman et al. (2018)
*Lemna valdiviana*	0.5 mg L^−1^ As(V), 7 days	Accumulator	Rhizofiltration	Souza et al. (2019)
*Pistia stratiotes*	1–4 days, 5–20 µM As(III)	Excluder	Rhizofiltration	de Campos et al. (2019)
*Pistia stratiotes*, *Spirodela polyrhiza*, *Eichhornia crassipes*	15 days	Accumulators	Phytofiltration	Rai (2019)
*Eichhornia crassipes*	3 days, 7 μM As	Accumulator	Rhizofiltration	de Souza Reis et al. (2020)
*Elodea canadensis*	15–250 µg As L^−1^ 72 h	Excluder	Rhizofiltration	Picco et al. (2019)
*Vallisneria natans*	14 days	Excluder	Rhizofiltration	Li et al. (2018b)
*Salvinia molesta*	0–20 µM As(III), 96 h	Accumulator	Phytofiltration	da Silva et al. (2018b)
*Eupatorium cannabinum*, *Dittrichia viscosa*, *Melilotus alba*, *Betula pubescens*, *Populus nigra*	11 mg L^−1^ As(V)	Excluders	Phytostabilisation	Gonzalez et al. (2019b)
*Dahlia pinnata* Cav	0–120 µM	Excluder	Potential phytostabilisation	Raza et al. (2019)

### Plant traits

Several model plants with potential use in phytoremediation are investigated for their biological responses to As excess and their As accumulation in plant parts (Table 1). Rice is of concern for grain As and one studied crop for the mechanisms of As uptake, distribution, and detoxification (Zhao 2020). As tolerance in As excluders is likely partly based on the suppression of high affinity phosphate/arsenate co-transport systems (Karimi et al. 2009, Karimi and Souri 2016).

#### Brassicaceae

*Isatis cappadocica* metallicolous (M) and non-metallicolous (NM) populations (Iran, temperate Asia) are claimed As accumulators, with potentially constitutive As chelation by thiols and PCs, and tolerance not based through deletion of high-affinity P/As co-transport (Karimi et al. 2009). Both NM and M populations are more resistant than other commercial Brassicaceae (e.g. broccoli, cabbage, and cauliflower), M plants being more As-tolerant than NM ones over 1.3 mM As. At 1825 mg As kg^−1^ (fading soil series with mine soil), *I. cappadocica* shoots displayed 350 mg As kg^−1^. Potential mechanisms, e.g. detoxification of oxidative stress, are discussed in Souri et al. (2020). Indian mustard (*Brassica juncea*, Gupta et al. 2009) and Ethiopian mustard (*B. carinata*, Irtelli and Nacari-Izzo 2008) can display high shoot As concentration, but their phenotype is not well established (Karimi et al. 2009). In Indian mustard cultivars with shoot As concentration ranging from 16 to 1138 mg kg^−1^ DW, 10,870 genes are differentially expressed mainly in reaction to stress, metabolic processes, transporter activity, and signal transduction (Thakur et al. 2019). Transcription regulator activity is up-modulated whereas many genes implied in photosynthesis, developmental processes, and cell growth are downregulated.

*Arabidopsis thaliana* was modified into an As accumulator by heterologously expressing PvACR3 in the athac1 background and knocking out the *HAC1* gene, AtHAC1 being an As reductase (Wang et al. 2018a). Muting the As reductase reduced As efflux into the medium (Zhang et al. 2018). For these transgenic plants, expression of the vacuolar As transporter ACR3 in the roots did not promote As(III) efflux into the medium, nor its vacuolar sequestration, but helped As loading into the vasculature and promoted translocation to the shoots. In transgenic *A. thaliana* and soybean, PvPht1;3 is expressed in stele cells and probably contributed to P/As translocation. Such PvPht1;3 expression raises As transfer and build-up in shoots, which may improve As phytoextraction in As-polluted soils.

#### Ferns

Several ferns (Pteridaceae), i.e. *Pteris vittata*, *P. cretica*, can tolerate and hyperaccumulate high As levels, without visible phytotoxicity symptoms. For sporophytes exposed to As(V), arsenate is absorbed by the roots, translocated via the xylem, and stored in the vacuoles of the leaf-like fronds as As(III) (Cai et al. 2019). *Pteris vittata* efficiently extracts As in low-P soils, increases in root growth and exudation helping to solubilise non-labile As and P from soils (da Silva et al. 2018a, Fig. 3). The *P. vittata* roots absorb arsenate via a PO_4_ transporter (PvPht1;3), while the aquaporin tonoplast intrinsic protein 4 (PvTIP4) eases the arsenite absorption. The PvACR2 As(V) reductase, the PvGRX5 glutaredoxin, and maybe other reductases are then converting arsenate to As(III) (Cai et al. 2019). The As(III) loading into the vacuoles of *P. vittata* gametophytes is facilitated by the PvACR3 As(III) transporter. Three genes, i.e. glyceraldehyde 3-phosphate dehydrogenase (PvGAPC1), glutathione S-transferase (PvGSTF1), and organic cation transporter 4 (PvOCT4), As-upregulated, are needed for As tolerance (Cai et al. 2019). The PvGAPC1 protein includes an uncommon active site having a lower affinity for phosphate than arsenate; PvOCT4 is present as puncta in the cytoplasm and PvGSTF1 displays As(V)-reductase activity. Arsenate, PvGSTF1, and PvGAPC1 are co-located. Once As(V) enters the cell, it would be converted into 1-arseno-3-phosphoglycerate by PvGAPC1. This one would be pumped into As-metabolising vesicles by the PvOCT4 protein and then hydrolysed to release As(V); this allows thereafter its reduction by PvGSTF1 to As(III) and vacuolar compartmentation. While phosphate limits As root uptake, phytate increases As absorption and development of *P. vittata* by regulating phosphate transporters, i.e. PvPht1:3 increased while PvPht1;1 decreased (Liu et al. 2018). The P and Si transporters mainly facilitate As absorption and its excess damages plant metabolism at various levels, e.g. impacts through oxidative stress, carbohydrate metabolism tightly connected to photosynthesis, and metabolic pathways coping with As-induced oxidative and nitrosative stresses. The PvPht1;3 gene from P*. vittata* complements a yeast P-uptake mutant and has a greater affinity and carriage capacity to As(V) than PvPht1;2 (Cao et al. 2019).

Vacuolar sequestration is one main mechanisms for plants to control excessive exposure to metal(oid)s. Angiosperms sequester PC–As(III) or GSH (glutathione)–As(III) conjugates in their root vacuoles (Zhao et al. 2010). PvACR3 occurs in the genomes of all the main plant lines but not in angiosperms, possibly explaining why they cannot accumulate or tolerate high As levels. Arsenic transporters are identified in *P. vittata*: a MIP (major intrinsic protein), PvTIP4;1, is mediating As(III) inflow into cells, whereas PvACR3 and PvACR3;1 intervene As(III) efflux into vacuoles (Yang et al. 2018). 824 transcripts are differentially expressed in As-stressed *P. vittata* ferns (Potdukhe et al. 2018). These genes are transcription factors and metal transporters, or are involved in chelator biosynthesis in line with absorption and accumulation mechanisms: e.g. cysteine-rich RLK, and ABC transporter G family member 26. Yan et al. (2019) have proposed a network consisting of six major transporter families, i.e. arsenical resistance protein Acr3 (ACR3), the major facilitator superfamily (MFS), the ABC superfamily, P-type ATPase, MIP, and nitrate transporter 3.1 (NRT3.1), two resistance pathways (i.e. GSH metabolism, notably Glutathione S-transferase (GST) and endoplasmic reticulum-associated protein degradation (ERAD) in roots, and a regulatory system for As hyperaccumulation—regulation mechanisms in *P. vittata* tissues following high acute As(III) and As(V) exposure.

#### As-(hyper)tolerant grasses and dicots

Cultivation of forage crops and perennial grasses may either reduce the human exposure to As while increasing the farmer incomes or promote the vegetation cover reducing the contaminant dispersion by natural agents. As-hypertolerant populations of *Holcus lanatus* are identified and assessed at several contaminated sites (Hartley-Whitaker et al. 2002; Mench et al. 2003a, b, 2010; Karczewska et al. 2013). At the acidic metal/As-polluted Jales mine tailings, the combined addition of Beringite (a fly ash containing modified aluminosilicates), iron grit and compost was most effective to change As phytoavailability and soil pH and, after 4 years, to sustainably improve the revegetation by As-excluder colonists, notably *Agrostis castellana* and *H. lanatus* (Bleeker et al. 2002, Fig. 3). *Rosmarinus officinalis* has a potential to phytostabilise As and metals in Mediterranean area (Affholder et al. 2014).

*Lolium multiflorum* var. *italicum*, *Secale cereale*, *Vicia villosa*, and *Trifolium pratense* were cultivated in an agricultural soil adjacent to a mining area after amendment with either pig manure or AMD sludge (AMDS) (Kim et al. 2018). Bioavailable soil As increased in pig manure-treated soil, due to desorption by dissolved organic matter, in both non-cultivated and cultivated soils, apart for *T. pratense*; in contrast, it fell down in all AMDS-treatments. To cultivate such excluders resulted in safe crops. Water stable aggregation was enhanced by these plants, but As phytostabilisation did not occur. In situ soil flushing (8 days, with monopotassium phosphate (KH_2_PO_4_) solutions) combined with cultivating *P. vittata* and *L. multiflorum* facilitated As removal from the rhizosphere soil, reaching 35% and 53%, respectively, which exceed the 9% removal rate from soil flushing alone (Yan et al. 2018). Some As excluders, aromatic plants from the Poaceae (e.g. Vetiver (*Chrysopogon zizanioides*), Lemon grass (*Cymbopogon flexuosus*), Palmarosa (*Cymbopogon martinii*), and Citronella (*Cymbopogon winterianus*), Lamiaceae (Ocimum, Mentha, Lavender, Salvia, and Rosemary), Asteraceae (Chamomile), and Geraniaceae (*Pelargonium* sp.) families, used for producing essential oils, can give financial returns and have a potential for phytomanaging As-contaminated soils as these non-food crops are reducing the hazard of food chain contamination (Pandey et al. 2019).

*Miscanthus x giganteus* was growing well during 2-year in potted soils collected at former military sites, i.e. Sliac, Slovakia and Kamenetz-Podilsky, Ukraine (Pidlisnyuk et al. 2019). Major part of the metal(loid)s remained in the roots, notably in year 2, and rather limited amounts moved to the shoots, foliar As concentrations being below detection limit. Both *Miscanthus sacchariflorus* A0104, and *M. sinensis* C0424 and C0640 exposed to As-spiked soil display As-excluder phenotype (Jiang et al. 2018). The biomass of *Phragmites australis* and *Arundo donax*, both being As excluders, increased in amended, As/metal-contaminated soils and soil amendments promoted aided phytostabilisation in the decreasing order: municipal solid waste compost (MSW-C) > Fe-rich water treatment residue (Fe-WTR) + MSW-C > Fe-WTR (Castaldi et al. 2018). At acidic soil pH (3.8), the highest As accumulation was recorded for plants grown on untreated soil. At neutral and alkaline soil pH, root As concentrations increased for compost-amended soils.

#### Macrophytes

Macrophytes are potentially useful for filtering As-contaminated effluents and water through their rhizosphere and root mats. In *Eichhornia crassipes* 3-day-exposed to 7 µM As, defense mechanisms against oxidative stress, enzyme activities related to S metabolism, and chelating substances are stimulated: the ATP sulphurylase (ATPS) activity increases in roots (de Souza Reis et al. 2020). Glutathione reductase (GR) activity in leaves and glutathione peroxidase (GSH‐Px) in roots decrease. Glutathione sulphotransferase (GST) activity is enhanced in roots, suggesting increased GSH conjugation to As, and limited in leaves, and γ‐glutamylcysteine synthetase (γ‐ECS) activity is higher in leaves, suggesting PCs synthesis.

The aquatic moss *Warnstorfia fluitans* can filtrate As(V) and As(III) from As-contaminated-water (Sandhi et al. 2018). Arsenic removal was faster in arsenite than arsenate solutions, optimum (80–90% within 2 h) being at pH 6.5 and 9.5 and at 20 and 30 °C, and at low oxygenation levels. No As net efflux process occurred in the water system except after 48 h in As(V)-treated medium at 30 °C. Most internal As, i.e. 95% in the As(V) and 85% in the As(III) treatments, was bound to the tissue.

Rhizofiltration of As by macrophytes is also documented for *Sagittaria montevidensis* (Demarco et al. 2019); water hyacinth (*E. crassipes*), water ferns (*Azolla* spp.), duckweeds (*Lemna* sp., *Spirodela* sp. and *Wolffia* sp.), hydrilla (*Hydrilla verticillata*), and water cresses (*Nasturtium officinale*, *N. microphyllum*) have a potential for phytofiltration (Rahman and Hasegawa 2011). Twenty macrophytes from an Italian wetland area affected by urban and industrial pollutants display high tolerance to metal(loid) excess and capacity for phytostabilisation (Bonanno et al. 2018). Bioconcentration factor (BCF) values (from sediment to roots) for As ranged from 0.02 (*N. officinale*, *Paspalum paspaloides*) to 0.25 (*E. crassipes*, *P. australis*); leaf/ root transfer factor (TF) values varied from 0.12 (*Cyperus longus*) to 1.32 (*Lemna gibba*). *Lemna* sp. can uptake high amounts of metal(loid)s with a relatively high biomass yield. Efficiency of *Lemna valdiviana* for As removal peaked between pH 6.3 and 7.0, with available phosphorus of 0.049 mmol P-PO_4_ L^−1^ and 7.9 mmol N-NO_3_ L^−1^, accumulating up to 1190 mg As kg^−1^ DW (Souza et al. 2019). *Eichhornia crassipes* (60%) was more efficient for As removal than *Pistia stratiotes* (water lettuce, 49%) and *Spirodela polyrhiza* (a duckweed, 37%) in a microcosm experiment (Rai 2019).

In *Elodea canadensis* plants exposed to 15 µg and 250 µg As L^−1^, foliar As concentration reached 0.34 and 0.4 mg As kg^−1^, and roots 0.34 and 4.3 mg As kg^−1^, as an excluder (Picco et al. 2019). These plants can filtrate As from tap water of an Argentine city located in an As-endemic area from 36 µg As L^−1^ to undetectable levels (10 ng As L^−1^). The submerged macrophyte *Vallisneria natans* was exposed to the binary As(III)/As(V) system (Li et al. 2018b). Total As and As(III) in water dropped within 3 days, while As(V) first increased slightly and then gradually decreased. About 1.2% DMA was detected at day 14. Both As(III) and As(V) were higher in roots than in leaves. In leaves, As(III) increased with the elapsed exposure time. The proportions of As(V) (28–40%) were lower than those of As(III) and arsenobetaine (AsB) was detected (0.5–1.9 mg kg^−1^) after day 7, suggesting As(V) reduction and As(III) methylation to AsB. In *Salvinia molesta* (an aquatic fern) exposed to 20 µM As(III) for 4 days at pH 6.5 floating leaves reached 103 mg As kg^−1^ (da Silva et al. 2018b).

In constructed wetlands (CW), high As removal is obtained with *Zantedeschia aethiopica* and *Anemopsis californica*, *Eleocharis macrostachya*, *Schoenoplectus americanus*, *Juncus effusus*, *Phragmites australis*, and *Echinodorus cordifolius* (Corroto et al. 2019). *Pistia stratiotes* plants were exposed in the 0–20-µM As(III) range for 4 days (de Campos et al. 2019). Root and shoot As concentrations peaked at 10 µM As(III) (i.e. 1120 and 31 mg kg^−1^ DW), displaying an excluder phenotype. At the lower As exposure, the biomass production was not affected; at 20 µM As(III), it decreased by 77%. Chlorosis, darkening, and reduction of the root system were mirrored by increased membrane damages and the contents of reactive oxygen species. Rhizofiltration using *Cyperus haspan* (A), *Juncus effusus* (B), and a mix of laterite and gravel (substrate as a control, (C)) in subsurface horizontal-flow constructed wetlands (CW) was assessed to decrease As concentration in the reverse osmosis residues in Buenos Aires province, Argentina, because As concentrations in drinking water for c.a. 10% of the population exceed the WHO threshold value (10 µg L^−1^) (Corroto et al. 2019). Arsenic removal ranged from 30 to 80% in the *J. effusus*-planted CW and between 10 and 40% using the *C. haspan-*planted CW. Arsenic concentration along CW was similar in the C and A treatments. The cumulative As mass was 62%, 34%, and 27% for A, B, and C treatments, respectively. During the elapsed time, *C. haspan* and *J. effusus* contributed between 12 and 67% and 22 and 87%, respectively. For *J. effusus*, the accumulation is higher than the translocation process (BCF 1.6 and TF 0.2), whereas for *C. haspan* both factors were analogous (1.1 and 1.0, respectively). In a pilot-scale CW filled with laterite soil (20–28% Fe by weight) and planted with *Colocasia esculenta*, water As concentration decreased by 89% in the planted CW as compared to 52% in the unplanted one (Vanlop et al. 2019). Arsenic was mostly located within the root zone, because of rhizo-stabilisation and the Fe-adsorbing process within the laterite soil. Its sorption increased with the elapsed time. CWs planted with *P. australis* (Mexico) displayed high As removal rate (73–83%), and metal(loid) amounts removed from the substrates were in decreasing order: Fe > Cu > As (Cortes-Torres et al. 2019).

#### Trees

Tree root systems are an advantage to colonise and remediate deep contaminated soil layers. Salicaceae, i.e. willows, poplars, is one option for biomass production on As-contaminated soils (Purdy and Smart 2008; Vamerali et al. 2009; Janssen et al. 2015; Jiang et al. 2015, Bart et al. 2016). Their high biomass may compensate for their moderate shoot metal(loid)-concentrations. In hydroponics, *S. viminalis x S. miyabeana* and *S. sachalinensis x S. miyabeana* hybrids were most tolerant to As excess (Purdy and Smart 2008). Mechanisms involved in As root uptake, storage in vacuole, potential transport through the plant, and As tolerance of *Salix purpurea* and *S. atrocinerea* are described in transcriptomic analyses (Yanitch et al. 2017; Navazas et al. 2019). In As-stressed *S. purpurea*, biosynthesis of phenylpropanoids is induced, with the increased production of tannins. In hydroponics (18 mg As L^−1^), a *S. atrocinerea* clone, from an As-contaminated brownfield, concentrates up to 2400 mg As kg^−1^ DW in roots and 25 mg As kg^−1^ DW in leaves. Roots reducing As(V) to As(III), As(III) predominates in roots and As(V) in leaves (Navazas et al. 2019). Since day 1, leaves and roots display de novo synthesis and increased non-protein thiols. To cultivate fast-growing willow shrubs, either in CWs or soils, can be a flexible and inexpensive solution to treat wood leaching containing metal(loid)s or polychlorinated dibenzo-dioxin/furan congeners (PCDD/Fs) generated at wood preservation sites (Fredette et al. 2019). *Salix miyabeana* ‘SX67’ was grown in three substrates irrigated with leachates containing increasing concentration of pentachlorophenol (PCP) and chromated chromium arsenate (CCA) over 12 weeks. The growing substrate affected willow ecophysiological responses and overall performance, leaf area being decreased with rising leachate concentration. Contaminants were stored in willow roots, but PCDD/Fs and Cu were also allocated to shoots.

*Populus nigra* seeds collected in the Loire Valley, France, were short-term cultivated on a potted soil from As/Pb mine tailings with three amendments, i.e. garden soil, compost and biochar, either alone or combined (Nandillon et al. 2019). The As concentration in the soil pore water (SPW) increased in all amended soils (18 to 416 times) notably with the compost treatment, which may promote As leaching. Seed germinated and plant grew only on amended soils, but adding biochar was less efficient. Poplar plantlets were As excluders, but the sustainable colonisation of such tailings by poplar populations remain to prove. Arsenic phytoextraction by four tree species, i.e. *Acer pseudoplatanus*, *Quercus robur*, *Betula pendula*, and *Ulmus laevis,* was evaluated in hydroponics (1 mM As(V), Budzynska et al. 2019a). The As accumulation peaked in *B. pendula* (BCF = 0.87) and *Q. robur* (BCF = 0.5). *Betula pendula* retained about 80% of As in its roots (excluder, TF = 0.2) whereas *Q. robur* allocated more than 60% of As in its shoots (TF = 1.6), which can be in hydroponics a sensitive behavior due to As excess as well as for *U. laevis* and *A. pseudoplatanus*. As(V) phytoextraction decreased root P and S concentrations in these tree species. The absorption of inorganic (As(III), As(V)) and organic arsenic (As-org) forms was then assessed with these tree species and two others, i.e. *Acer platanoides*, and *Tilia cordata*, in a pot experiment with As-contaminated mining sludge (Budzynska et al. 2019b). Total As was mainly stored in the roots of all these tree species, which were generally thinner, shorter and/or black after the experiment. The As(III) and As(V) concentrations peaked in the *A. pseudoplatanus* and *A. platanoides* roots (174 and 420 mg kg^−1^, respectively). Relatively high As(III) concentrations (in mg kg^−1^) were recorded in the *B. pendula* shoots (12) and As(V) in the shoots of *U. laevis* and *A. pseudoplatanus* (77 and 70). With some exceptions, As-org (present in mining sludge in low concentration) predominated in the tree organs. Influence of As forms, i.e. As(III), As(V), and DMA was assessed with *A. platanoides* in hydroponics (Budzynska et al. 2018). The exposure to particular As forms in single, double, and triple experimental systems decreased the seedling biomass. Negative symptoms depended on arsenicals and their concentration in solution, ranging from slight visible changes (inorganic compounds separately or jointly), through smaller and discolored leaves (DMA exposure), and finally to their withering (high DMA excess). Changes in color and shape for root systems exposed to arsenical combination occurred, despite seedling biomass were not affected. Root, stem, and foliar concentrations plateaued at 590, 70, and 140 mg As kg^−1^ DW, respectively, under different combinations, showing an excluder phenotype. The highest BCF values reached 10.8 for root systems exposed to 0.06 mM of As(V) and DMA, while the highest TF value (1.0) was for 0.6 As(V) plus 0.06 mM DMA.

#### Halophytes

Phytomanagement of contaminated soils is of concern also in area affected by salinity (Sghaier et al. 2019). *Atriplex atacamensis*, a perennial shrub from Northern Chile occurring on As-contaminated area, may experience transient flooding conditions. This As excluder was exposed to either 50 µM As(III) or As(V) (Vromman et al. 2018). As(III) decreased plant development, stomatal conductance, and photosystem II efficiency while As(V) did not. Root As concentration peaked in reaction to As(III) excess in contrast to As(V). As(III) oxidation may occur because As forms are detected in roots for each treatment. Over 40% of As was sorbed to the cell wall in the As(V)-exposed roots whereas this rate decreased in the As(III)-exposed ones. Total As and its cell wall-bound fraction in leaves were similar after As(V) and As(III) exposure. Non-protein thiols peaked in response to As(V) excess in comparison to As(III), whilst ethylene synthesis was only enhanced in As(III)-exposed plants. *Kosteletzkya pentacarpos* was cultivated on a column device allowing leachate harvest, on a metal(loid)-spiked soil (i.e. 6.5 mg Cd, 75 mg As, 200 mg Zn, and 300 mg Pb kg^−1^ DW) and irrigated with salt water (final soil electrical conductivity 5.0 mS cm^−1^) (Zhou et al. 2019). Salinity decreased bioavailable soil As and shoot As concentration (0.7–1 mg As kg^−1^, excluder).

*Halogeton glomeratus* seeds, from arid regions in Northwest China, were sown in metal(loid)-contaminated saline soil plots (Li et al. 2019a). In year 1, total salt yield extracted from plants was 2105 kg ha^−1^, and salt concentration was 1.61 g As kg^−1^. Seeds contained 0.26 mg As kg^−1^ and their oil content was 19% with 91% of unsaturated fatty acids.

### Field survey and case studies

Out of 20 native species from the Baoshan mining area (China, total soil As: 125–6656 mg kg^−1^), *Pteris ensiformis* accumulated 1091 mg As kg^−1^ in its shoots, with potential use for As phytoextraction (Pan et al. 2019); *Boehmeria nivea* shoots, usable for textile fibers, reached 701 mg As kg^−1^. In both cases, high root-to-shoot transfer factor and unexpected high shoot Pb concentrations however may mirror foliar exposure. Several As excluders, i.e. *Dicranopteris linearis, Histiopteris incisa*, *Nephrolepis hirsutula*, *Pinus* sp., *Thysanolaena latifolia*, and *Melastoma malabathricum*, and As-accumulators, i.e. *Pityrogramma calomelanos* (210 mg As kg^−1^) and *P. vittata*, were identified nearby the Lepanto As–Cu–Au mine in the Philippines, being options to post-mining rehabilitation (Claveria et al. 2019). Many herbaceous plant species growing in mining area, e.g. *Agrostis castellana*, *Rumex acetosella*, can display high root As concentration (> 200 mg kg^−1^), being candidates for phytostabilisation (Otones et al. 2011).

One option to phytoextract As from contaminated soils is to use As-hyperaccumulating ferns from the *Pteris genus*, e.g. *P. vittata*, *P. cretica*, *P. longifolia*, and *P. umbrosa*, and other ones, e.g. *Pityrogramma calomelanos* var*. austroamericana* (gold dust fern) and *P. calomelanos* (silver fern) (Francesconi et al. 2002; Niazi et al. 2012). *Pteris vittata* prefers to grow in alkaline soils, shady area and in warm, humid climates. However, it can be grown outside in mild-Atlantic climate (SW France), and protect from strong frost under cold greenhouse or by mulching.

As hyperaccumulation is a constitutive trait for *P. vittata*, but M populations from As-contaminated soils differ from NM ones for As accumulation (Wu et al. 2018). Fern NM populations were collected from two Chinese uncontaminated sites, i.e. ZD and NN, and M ones in As and Pb/Zn mining and/or smelting sites, i.e. SG and GY. Both NM populations display higher As(V) and As(III) uptake than the M ones. Arsenate reductase activities in roots peaked in the NM populations. Root exudates from the NN and GY populations contained similar organic acid patterns, dominated by oxalic acid (> 67%) plus malic and succinic acids. For oxalate, the NN population released 4.2 times more than the SG one. The NN root exudates mobilised more As from polluted soils than the SG ones, oxalate being the most efficient to extract As.

In hydroponics (24 days) and in the presence of Pb and Cd, the temperate zone fern *P. multifida* (able to tolerate low temperatures from 5 to − 4.6 °C) removed 50% of As(III) (Rahman et al. 2018). Frond As concentration was higher than in other plant parts whereas Cd and Pb concentrations peaked in roots and rhizome. In potted spiked soils, As concentration reached 1200 and roughly 250 mg kg^−1^ in the rhizome and fronds of *P. multifida*, whereas the frond As concentration of the tropical zone fern *P. vittata* was 2100 mg kg^−1^. Co-accumulation of metal(loid)s by *P. vittata* is questionable. Two Chinese *P. vittata* populations, one from a Sb smelting area (total soil As: 147) and one from a Pb–Zn mining area (total soil As: 572), and one As-excluder *Holcus lanatus* population were cultivated on a Sb/As-polluted soil (Wan et al. 2018). The fern displayed high As- but limited Sb-accumulating capacity: shoot As and Sb concentrations culminated at 455 and 26 mg kg^−1^, respectively. At day 28, the Sb and As concentrations in the soil solution were respectively decreased by 22% and 36% in the fern treatments. In contrast, the Sb and As accumulation by *H. lanatus* shoots was limited. In *P. vittata*, As(V) was converted to As(III), which dominated in shoots, but reduction of antimonate to antimonite was limited (with > 90% of shoot Sb existing as antimonate). The fern M population showed 35% higher As uptake than the NM one. Both populations did not differ for Sb accumulation. In contaminated soils dominated by Cu excess from a wood preservation site, *P. vittata* growth is affected and As-phytoextraction limited (Mench unpublished). For *P. calomelanos* plants exposed at 1 mM As, 90% of the As absorbed was accumulated in shoots, and no As stress symptoms were visible on plant parts (Campos et al. 2018). At higher exposure (10 and 30 mM As), As uptake by roots was mainly translocated into the shoots (81–74%), with marginal and apical necroses on pinnae, damages being mainly in the secondary veins and adjacent cells. In the As-stressed roots, tenuous alterations were identified, i.e. separation of border-like cells and presence of granular substances in cortical cells.

Several field trials are cited by Jiang et al. (2015): *P. vittata* and *P. cretica* in southwest England (Gray et al. 2005), low shoot DW yield (*P. vittata*, 0.76 t DW ha^−1^) being the main drawback; *P. vittata* assessment at 21 As-contaminated sites in England (Shelmerdine et al. 2009) demonstrating that the As amount phytoextracted generally fell down as total soil As expanded and pointing out the low fern yield; and a Chinese trial (2t DW ha^−1^, Chen et al. 2006). At a former CCA-contaminated site in Florida, total topsoil As was decreased from 190 to 140 mg kg^−1^ following a 2 year-cultivation of *P. vittata*. (Kertulis-Tartar et al. 2006). Using this fern, 8 years would be required to decrease the acid-extractable soil As from 80 to 40 mg kg^−1^ (US-EPA limit) at an EPA Superfund site (Salido et al. 2003). The *P. vittata* capacity to phytoextract As decreases after several consecutive frond harvests and this fern species did not well regrow in the plots due to competition with weeds (Reichmann et al. 2004 cited in Niazi et al. 2012; Mench et al. 2014).

Silverback fern, *P. calomelanos*, is able to better prosper on tropical As-polluted soils than *Pteris* sp. (Clemens and Ma 2016). It was cultivated in both greenhouse and field trials in an As-polluted area of the Ron Phibun District, Thailand (Jankong et al. 2007). Rhizosphere bacteria and fungi were isolated from the fern roots. P fertiliser and rhizobacteria increased plant biomass and As accumulation, thus As phytoextraction. Rhizofungi decreased plant As concentration but enhanced plant biomass. Gold dust fern and Chinese brake fern were compared at a disused As-contaminated cattle-dip site for their As-phytoextraction capacities over 27 months (Wollongbar, NSW, Australia; Niazi et al. 2012). The frond DW yield, As concentration and As uptake were higher in the Gold dust fern than in *P. vittata*, at all harvests (i.e. 10, 22, and 27 months). Gold dust fern phytoextracted 25.4 kg As ha^−1^ (cumulative over three harvests), 2.65 times more than *P. vittata* (9.7 kg As ha^−1^), corresponding to 1.7–3.9% and 0.53 − 1.5% of total topsoil As. To assess P/As interaction, *P. vittata* was cultivated in two sandy polluted soils (C soil from an As-treated wood facility and D soil from a cattle-dipping vat, 129 and 26.7 mg As kg^−1^) over 5 years and during 10 harvests, under P-sufficient (P-fertiliser) and P-limiting (phosphate rock) conditions (da Silva et al. 2018a). Frond biomass production and As removal peaked for the 9th (62–64 and 35–63 g As plant^−1^) and 10th harvests (58–60 and 52–57 g As plant^−1^) for C and D soils, even though As concentration in fronds dropped. Soil As phytoextracted averaged 7–10% per harvest during the 1–6th harvests; it decreased to 0–3% during the 7–10th harvests for D and C soils. All soil As fractions, except the residual one, were concerned by plant uptake. Highest decrease occurred in the amorphous fraction of the C soil (64–66%) and in the crystalline fraction of the D soil (50–86%). Soil As concentrations decreased respectively by 37–47% from 26.7 and 129 to 16 and 69 mg kg^−1^ for the D and C soils. Non-labile As was efficiently mobilised by *P. vittata* under P-limiting conditions without affecting its As depletion.

Soil pH is one major driving force for As phytoextraction. In a field survey, *P. vittata* and *P. calomelanos* only occurred in As-polluted areas with soil pH 7.2–8.8 and 2.3–4.2, respectively (Anh et al. 2018). Both fern species were further grown in potted soils spiked with 300 mg As kg^−1^ with soil pH set at 5.1, 7.2, and 9. Silverback fern subsisted at these three soil pH and showed the highest frond As concentration and soil As phytoextraction at soil pH 5.1. In contrast, all *P. vittata* plants perished at this soil pH. At soil pH of 7.2 and 9, *P. vittata* displayed higher frond As concentration, shoot biomass, and shoot As removal than *P. calomelanos*. For alkaline soil (pH 7.8) spiked with increasing As levels, *P. vittata* exhibited higher life time, shoot biomass, As tolerance, and phytoextraction than *P. calomelanos*.

## Conclusions and perspectives

The evidences so far available indicate the exploitation of the microbial As(III) oxidation as the most promising application for water treatment and bioremediation purposes. To date, the As transformation processes and the associated high microbial and functional diversity have been broadly studied and described. Only few investigations have however tested the biological As(III) oxidation process under exploratory settings mimicking real situations (e.g. long-term experiments managed at large scale and/or in water treatment plant) and this has strongly impacted on the further development of bio-based technologies. Consequently thereof, the field applicability of As microbiological remediation processes in combination with conventional methods was not fully exploited and evaluated so far. Overall, to employ autotrophic As(III) oxidisers may be favoured as the process does not required the addition of any organic carbon sources. However, this metabolism has been till now exclusively found in microorganisms isolated from extreme environments. Nevertheless, several bench scale studies showed the efficient exploitation of heterotrophic As(III) oxidation in bioremediation strategies. This potential deserves to be further investigated and assessed in systems at higher scale. Reductive microbial bio-processes also present interesting potential in terms of As removal from water, in particular for mine and industrial polluted streams. The anaerobic bioreactors could be judiciously combined with downstream aerobic oxidative and/or phytoremediation steps, for removing the residual dissolved As and organic carbon, as shown in the example of Trail site (Al et al. 2011). As observed with other pollutant classes, the use of native bacteria is surely a feasible option for bioremediation purpose due to their high compatibility with the environment and tolerance to toxicity. Diversely, bioaugmentation with specialised bacteria implies the analysis of additional constrains that may be costly (e.g. the pre-cultivation and addition of highly concentrated microbial cultures) and may negatively impact the As transformation rate (e.g. the limited adaptation and persistence of the added microorganisms in the highly competitive environment).

Recent methods of genomics, such as DNA sequencing and transcriptome analysis, are at the interface between molecular biology and ecology. When they are applied to environmental issues, they go beyond the simple description of organisms present in ecosystems. They make indeed possible to characterise microbial communities, which are sometimes complex and which can shelter organisms recalcitrant to conventional cultural methods. Combined with functional approaches such as metaproteomics, metabolomics, and stable isotope probing (Fischer et al. 2016; Musat et al. 2016; Vogt et al. 2016; Zuñiga et al. 2017), these various methods can provide an overall vision of the ecosystem structure and functioning. Moreover, with the sequencing depth that these recent technologies are able to do, access to the less-depicted species, the so-called rare biosphere, becomes possible. Such an opportunity to characterise microbial communities quickly and cost-effectively could be particularly useful in monitoring bioremediation processes in As-contaminated environments, thus avoiding changes in parameters that would compromise the efficacy of bioremediation (Lovley 2003; Stenuit et al. 2008; Techtmann and Hazen 2016). Nevertheless, these approaches must be combined with laboratory and field experiments. In addition, they often require the development and optimisation of reproducible and efficient biological sampling and extraction methods. Finally, storing, exchanging and analysing the massive data amounts generated by high-throughput sequencing methods require the implementation of robust new computing methods, much more complex than those required by conventional statistical analyses (Pasolli et al. 2016; Li et al. 2017). The resulting studies must also include a set of complementary data, called metadata. Collected for each genome or metagenome studied, they must allow appropriate data exploitation (Satinsky et al. 2013), as specified by the Genomic Standard Consortium (Yilmaz et al. 2011).

Several practices are developed to enhance or reduce As (phyto)availability depending on the (bio)remediation purposes and pollutant linkages. Use of chelating agents in combination with plants can promote either As phytostabilisation in excluder roots or bioavailable As stripping by accumulators, but it is controversial as metal(loid)-chelates may be lixiviated and contaminate the groundwater. Soil amendments, e.g. Fe/Mn bearing phases, alkaline silicon slags, are investigated to regulate root As exposure (Kumpiene et al. 2019, 2021). Effectiveness of composts and biochars in reducing As bioavailability depends on many factors including the release of dissolved organic matter, Fe/Mn oxide content, dosage rate, etc. (O’Connor et al. 2018). Si-based fertiliser can mitigate As accumulation in rice (Zhao et al. 2010; Zhao 2020). Soil fertilisation (e.g. N, calcium phosphate) and inoculation with arbuscular mycorrhizal fungi (e.g. *Funneliformis mosseae, Glomus mosseae*) can increase fern biomass but with few or no effect on frond As removal (due to dilution in the biomass), whereas soluble P addition may desorb As from the soil bearing phases and promote its leaching (Matzen et al. 2020). The selection of plant species and genotypes can be used to produce safe crops regarding As (Allevato et al. 2019). The genetic engineering is also explored to improve As excluders or (hyper)accumulators (Clemens and Ma 2016; Zhang et al. 2018; Allevato et al. 2019; Zhao 2020). Bioaugmentation with Plant Growth Promoting Bacteria (PGPB) and endophytic bacteria can promote root As uptake and plant growth, and thus As phytoextraction. Intercropping and co-cropping of As-excluder cash crops with As-(hyper)accumulator ones is an option for maintaining agricultural production and harvesting valuable biomass during the phytoextraction of labile fraction of soil As in excess. Phytomanagement-borne biomass is a resource and not a waste. Combustion is becoming a past option (Nzihou and Stanmore 2013). Research trends focus on optimising the processing of such biomass in various ways. It can be converted into valuable platform-chemical compounds, bio-active products, biogas/syngas, bio-oils and biofuels (Carrier et al. 2012; Cai et al. 2021, Wang et al. 2021). (Hyper)accumulator biomass can be converted into biocatalysts. Ethanol extraction with anaerobic digestion is an option mobilising As from *P. vittata* biomass and producing methane (da Silva et al. 2019a, b). Solid residues can be processed by pyrolysis, producing biochars, and other technologies. Thermochemical processes, e.g. gasification and pyrolysis, can provide syngas and bio-oil products useable for heat and electricity generation and biosourced chemistry (He et al. 2019). Cai et al. (2021) extracted phenolic compounds from *P. vittata* biomass. Vegetal fibers and powders to reinforce bio-sourced plastics and cements are other processes to be explored.

Depending on the type of As-contaminated site and future land use, the science front (in a holistic approach) includes: to continue to identify relevant plant species and microorganisms regarding soil As phytomanagement, to focus on molecular mechanisms of As phytomanagement (Thakur et al. 2020), promotion of mesofauna and bacterial communities through agricultural practices such as the permaculture (especially to facilitate rooting, water and nutrient uptake), (phyto)management of other contaminants combined with the soil As contamination (notably the case-studies of soil As/Cd co-contamination (Zhao 2020) and As/persistent organic pollutants), use of agro-ecology for As-contaminated agricultural areas, biomonitoring of the food chain transfer (Grignet et al. 2020), use of nanoparticles to reduce As exposure, and development of the (phyto-borne)-biomass-processing technologies.

Some phytomanagement gaps can be complemented by bioremediation (Roy et al. 2015), as microbes can: carry out the bioremediation out of the root zone and for groundwater; reduce bioavailable soil As, allowing plant colonists to cover the soil and to initiate a complementary phytoremediation; enhance contaminant removal (or xenobiotic dissipation) by promoting plant growth (e.g. atmospheric N fixation, mineral solubilisation and release of nutrients, production of plant growth regulators such as auxins, gibberellins and cytokinins, decrease of ethylene synthesis by 1-aminocyclopropane-1-carboxylate (ACC) deaminase, and challenging of pathogenic bacteria) and changing arsenical speciation; processing of plant biomass by anaerobic digestion etc. The use of transgenic plants and microbes is not addressed here but discussed elsewhere (Roy et al. 2015; Thakur et al. 2020; Zhao and Wang 2020) and currently not applicable in remediation strategies in Europe.

Public access to thousands of metagenomic samples, for example, from sites such as EBI metagenomics (Mitchell et al. 2017), associated with large data mining and analysis algorithms, and metabolic modeling methods is a real opportunity to better comprise how the various constituents of an ecosystem can work together in response to the biotic and abiotic factors of the environment. Rather than a simple inventory of biological objects, such a descriptive analysis can allow to answer questions such as: how do the concerned organisms work, what is their spatial and temporal distribution, what are the adaptive and even evolutionary processes involved and what are the metabolic interactions they may develop. In particular, such an integrated frame of the metabolic functions exerted by microbial communities should provide a better knowledge of the microbial processes at work in the biological treatment of As-contaminated water. Combined with the use of appropriate predictive models, this understanding should allow an optimal use of microorganisms and their properties for developing new biotechnological applications in the bioremediation field of As-contaminated soils and waters. They could also contribute to improve the functioning of existing bio-treatment processes and to better control and stabilise their long-term efficiency. The proof of the stability of biological activities in continuously fed treatment plants would help to increase the applicability of As bioremediation options and their acceptance as robust low-cost technologies by the economic sector. The biological data generated by metagenomics approaches could also be a source of information to propose and test the validity of bioindicators, potentially useful for monitoring the bioremediation processes or to assess the As bioavailability. Moreover, extended microbial metagenomic analyses of the different environmental compartments, i.e. soil, water, and rhizosphere soil, and the different plant parts will contribute to the development of As bioremediation processes involving cooperation between bacteria, fungi, and plants.

## Data Availability

Not applicable.
