# Correction to: Water and soil contaminated by arsenic: the use of microorganisms and plants in bioremediation

**DOI:** 10.1007/s11356-021-18056-3

**Published:** 2021-12-14

**Authors:** Philippe N. Bertin, Simona Crognale, Frédéric Plewniak, Fabienne Battaglia‑Brunet, Simona Rossetti, Michel Mench

**Affiliations:** 1 Génétique Moléculaire, Génomique Et Microbiologie, UMR7156 CNRS - Université de Strasbourg, Strasbourg, France; 2 Water Research Institute, National Research Council of Italy, (IRSA - CNR), Rome, Italy; 3 Environmental Biogeochemistry and Water Quality Unit, BRGM, Orléans, France; 4 Univ. Bordeaux, INRAE, BIOGECO, F‑33615 Pessac, France

**Correction to: Environmental Science and Pollution Research**

10.1007/s11356-021-17817-4

The article Water and soil contaminated by arsenic: the use of microorganisms and plants in bioremediation written by Philippe N. Bertin, Simona Crognale, Frédéric Plewniak, Fabienne Battaglia‑Brunet, Simona Rossetti and Michel Mench, was originally published electronically on the publisher’s internet portal on 16 June 2021 without open access. With the author(s)’ decision to opt for Open Choice the copyright of the article changed on 2 December 2021 to © The Author(s) 2021 and the article is forthwith distributed under a Creative Commons Attribution 4.0 International License, which permits use, sharing, adaptation, distribution and reproduction in any medium or format, as long as you give appropriate credit to the original author(s) and the source, provide a link to the Creative Commons licence, and indicate if changes were made. The images or other third party material in this article are included in the article’s Creative Commons licence, unless indicated otherwise in a credit line to the material. If material is not included in the article’s Creative Commons licence and your intended use is not permitted by statutory regulation or exceeds the permitted use, you will need to obtain permission directly from the copyright holder. To view a copy of this licence, visit http://creativecommons.org/licenses/by/4.0.

The Original article has been corrected.

